# Analysis of hereditary cancer syndromes by using a panel of genes: novel and multiple pathogenic mutations

**DOI:** 10.1186/s12885-019-5756-4

**Published:** 2019-06-03

**Authors:** Georgios N. Tsaousis, Eirini Papadopoulou, Angela Apessos, Konstantinos Agiannitopoulos, Georgia Pepe, Stavroula Kampouri, Nikolaos Diamantopoulos, Theofanis Floros, Rodoniki Iosifidou, Ourania Katopodi, Anna Koumarianou, Christos Markopoulos, Konstantinos Papazisis, Vasileios Venizelos, Ioannis Xanthakis, Grigorios Xepapadakis, Eugeniu Banu, Dan Tudor Eniu, Serban Negru, Dana Lucia Stanculeanu, Andrei Ungureanu, Vahit Ozmen, Sualp Tansan, Mehmet Tekinel, Suayib Yalcin, George Nasioulas

**Affiliations:** 1 Genekor Medical S.A, Athens, Greece; 2 Theagenio Anticancer Hospital, Thessaloniki, Greece; 3 Oncology Department, Athens Naval and Veterans Hospital, Athens, Greece; 4 Euroclinic Group, Athens, Greece; 5 Attikon University Hospital, Athens, Greece; 6 Athens Medical Center, Athens, Greece; 7 Euromedica General Clinic of Thessaloniki, Thessaloniki, Greece; 8 Metropolitan Hospital, Athens, Greece; 9 Papageorgiou Hospital, Thessaloniki, Greece; 10 IASO, General Maternity and Gynecology Clinic, Athens, Greece; 11 Spitalul Sfantul Constantin Brasov, Brasov, Romania; 12 Institutul Oncologic Prof. Dr. I. Chiricuta, Cluj, Romania; 13 University of Medicine and Pharmacy of Timisoara, Timisoara, Romania; 14 Institutul Oncologic Bucuresti, Bucuresti, Romania; 15 Amethyst Radiotherapy Cluj-Napoca, Cluj, Romania; 16 Faculty of Medicine Istanbul University, Istanbul, Turkey; 17 Tansan Oncology, Istanbul, Turkey; 18 Private Practice, Fulya Sisli, Turkey; 19 Private Practice, Kavaklidere, Turkey

**Keywords:** Next generation sequencing, Cancer susceptibility genes, Breast cancer, Hereditary cancer, *BRCA1* & *BRCA2*, Multigene panels, Pathogenic variant, Large genomic rearrangement, Genetic testing

## Abstract

**Background:**

Hereditary cancer predisposition syndromes are responsible for approximately 5–10% of all diagnosed cancer cases. In the past, single-gene analysis of specific high risk genes was used for the determination of the genetic cause of cancer heritability in certain families. The application of Next Generation Sequencing (NGS) technology has facilitated multigene panel analysis and is widely used in clinical practice, for the identification of individuals with cancer predisposing gene variants. The purpose of this study was to investigate the extent and nature of variants in genes implicated in hereditary cancer predisposition in individuals referred for testing in our laboratory.

**Methods:**

In total, 1197 individuals from Greece, Romania and Turkey were referred to our laboratory for genetic testing in the past 4 years. The majority of referrals included individuals with personal of family history of breast and/or ovarian cancer. The analysis of genes involved in hereditary cancer predisposition was performed using a NGS approach. Genomic DNA was enriched for targeted regions of 36 genes and sequencing was carried out using the Illumina NGS technology. The presence of large genomic rearrangements (LGRs) was investigated by computational analysis and Multiplex Ligation-dependent Probe Amplification (MLPA).

**Results:**

A pathogenic variant was identified in 264 of 1197 individuals (22.1%) analyzed while a variant of uncertain significance (VUS) was identified in 34.8% of cases. Clinically significant variants were identified in 29 of the 36 genes analyzed. Concerning the mutation distribution among individuals with positive findings, 43.6% were located in the *BRCA1/2* genes whereas 21.6, 19.9, and 15.0% in other high, moderate and low risk genes respectively. Notably, 25 of the 264 positive individuals (9.5%) carried clinically significant variants in two different genes and 6.1% had a LGR.

**Conclusions:**

In our cohort, analysis of all the genes in the panel allowed the identification of 4.3 and 8.1% additional pathogenic variants in other high or moderate/low risk genes, respectively, enabling personalized management decisions for these individuals and supporting the clinical significance of multigene panel analysis in hereditary cancer predisposition.

**Electronic supplementary material:**

The online version of this article (10.1186/s12885-019-5756-4) contains supplementary material, which is available to authorized users.

## Background

Hereditary cancer predisposition syndromes are responsible for approximately 5–10% of all diagnosed cancer cases [1, 2]. Identifying those cases is important both for the patient and at risk relatives, with clinical management implications both for affected and unaffected individuals. In affected patients, genetic determination of the inherited cause of the diagnosis can guide surgical management and in some cases systemic treatment. Furthermore, identification of the underlying syndrome can guide a personalized follow-up program, both for the patient and at-risk relatives, in order to incorporate surveillance and prevention strategies of secondary malignancies associated with the specific syndrome.

In the past, single-gene analysis of specific high risk genes was used for the determination of the genetic cause of cancer heritability in certain families. The selection of genes was largely based on personal and family history of the individual and included mainly the *BRCA1* and *BRCA2* genes for families with a breast/ovarian cancer history, the DNA mismatch repair (MMR) genes, *MLH1, MSH2* and *MSH6,* for families suspected to have Lynch Syndrome and the *APC* gene in patients with Familiar Adenomatous Polyposis (FAP). Today, the advent of Next Generation Sequencing (NGS) has allowed for multi-gene panel analysis, an approach now widely used in clinical practice for the identification of individuals with an inherited predisposition to cancer [3, 4]. These multi-gene panels usually include high and moderate penetrance genes and in many cases some low or of yet unknown risk genes. An increasing number of families are currently analyzed by the use of such panels. Thus, data concerning their contribution to cancer risk is constantly increasing, allowing a more accurate penetrance stratification. In the present study multigene NGS analysis was carried out in consecutive individuals referred to our laboratory. The aim of this analysis was the identification of cancer-susceptibility significant variants and the assessment of the applicability and utility of such an analysis for these individuals.

## Methods

### Study group

Individuals who were referred to our center for genetic testing with a hereditary cancer panel between June 2014 and March 2018 were evaluated. All samples were collected from the referring physicians during this study. As this study took place in a private diagnostic laboratory, subjects were not selected by strict inclusion criteria for genetic analysis. All individuals were informed about the significance of molecular testing, provided information about their personal and family history and have signed an informed consent form prior to molecular genetic testing and permission for the anonymous use of their data for research purposes and/or scientific publications. Information on demographics, clinical history, and family history of cancer was collected from test requisition forms, and pedigrees were provided by ordering clinicians at the time of testing.

### Gene selection

NGS analysis of hereditary cancer susceptibility genes was performed using two different gene panels. The genes analyzed were selected based on their association to hereditary cancer predisposition. In the majority of cancer syndromes the mode of inheritance is dominant. Thus, a single pathogenic variant in heterozygosity in one of these genes may be the causative reason of cancer predisposition. Several of these genes also have autosomal recessive inheritance, or result in clinically distinct autosomal recessive conditions. *BRCA2*, *BRIP1*, *PALB2*, and *RAD51C* are associated with Fanconi anemia. *ATM* and *MRE11A* are associated with ataxia-telangiectasia and ataxia-telangiectasia-like disorder (ATLD), respectively. *MLH1*, *MSH2*, *PMS2*, and *MSH6* are associated with constitutional mismatch repair deficiency (CMMR-D). *MUTYH* is associated with MUTYH-associated polyposis (MAP). *NBN* and *RAD50* are associated with Nijmegen breakage syndrome and Nijmegen breakage syndrome-like disorder (NBSLD), respectively. The majority of patients who required hereditary cancer testing had a personal or family history of Breast and/or Ovarian cancer and therefore, the vast majority of genes analyzed in this study are associated with increased risk of Breast and/or Ovarian cancer. In addition, the genes were further classified as high, moderate/intermediate or low penetrance genes based on their relative risk for cancer development that they confer to pathogenic variant carriers. High penetrance (or high risk) genes are considered those which when mutated, confer a high Relative Risk of cancer development (greater than 4 times the risk of the general population). Moreover, they are included in guidelines for cancer predisposition testing and specific clinical management recommendations for patients carrying pathogenic variants have been formulated by large working groups [5–7]. Pathogenic variants in moderate penetrance (or moderate risk) genes confer a 2–4 times risk of cancer development compared to the general population. Low penetrance/risk genes are those related to less than 2 times risk of cancer or those with limited or yet insufficient data available concerning their association and magnitude of cancer risk. Although this categorization is constantly altered in reflection to the accumulated clinical information, based on the latest published data [3, 5–10], the genes analyzed are summarized in Table 1.

**Table 1:** List of Genes analyzed by the Hereditary Cancer panels and their association with various cancer types and syndromes

Gene^a^	Transcript	Breast	Ovarian	Colorectal	Endometrial	Melanoma	Pancreatic	Gastric	Prostate	Endocrine	Other	Associated Syndrome
High Risk (***)												
*APC*	NM_000038.5			***			*	*		*	*	Familiar Adenomatus Polyposis (FAP)
*BMPR1A*	NM_004329.2			***				**			*	Juvenile Polyposis Syndrome (JPS)
*BRCA1*	NM_007294.2	***	***				*		**		*	Hereditary Breast and Ovarian Cancer Syndrome (HBOC)
*BRCA2*	NM_000059.3	***	**			*	*		**		*	Hereditary Breast and Ovarian Cancer Syndrome (HBOC)
*CDH1*	NM_004360.4	***						***				Hereditary Diffuse Gastric Cancer (HDGC)
*CDK4*	NM_000075.3					***						
*CDKN2A*	NM_000077.4					***	*					Familial Atypical Multiple Mole Melanoma Syndrome, Melanoma-Pancreatic Cancer Syndrome
*EPCAM*	NM_002354.2	*	*	***	**		*	*	*		*	Lynch Syndrome (LS)
*MEN1*	NM_000244.3						*			***		Multiple Endocrine Neoplasia Type 1
*MLH1*	NM_000249.3	*	**	***	**		*	*	*			Lynch Syndrome (LS)
*MSH2*	NM_000251.2	*	**	***	**		*	*	*			Lynch Syndrome (LS)
*MSH6*	NM_000179.2	*	*	***	***		*	*	*			Lynch Syndrome (LS)
*MUTYH*	NM_001128425.1	*		***	*			*			*	MUTYH-associated polyposis (MAP)
*PALB2*	NM_024675.3	***	*				*		*			Fanconi anemia (FA-N) (recessive)
*PMS2*	NM_000535.5		*	***	***		*	*	*		*	Lynch Syndrome (LS)
*PTEN*	NM_000314.4	***		*	*	*				**	**	Cowden Syndrome (CS)
*RET*	NM_020975.4									***		Multiple Endocrine Neoplasia Type 2
*SMAD4*	NM_005359.5			***				**				Juvenile Polyposis Syndrome (JPS)
*STK11*	NM_000455.4	***	*	**	*		**	**				Peutz–Jeghers Syndrome (PJS)
*TP53*	NM_000546.5	***	*	**	*	*	**	**	*			Li–Fraumeni Syndrome (LFS)
*VHL*	NM_000551.3						*			*	***	Von Hippel-Lindau Syndrome
Moderate Risk (**)												
*ATM*	NM_000051.3	**					*		*			Ataxia-Telangiectasia (recessive)
*BRIP1*	NM_032043.2	*	**						*			Fanconi anemia (FA-J) (recessive)
*CHEK2*	NM_007194.3	**	*	*					*			
*NBN*	NM_002485.4	**							*			Nijmegen Breakage Syndrome (NBS)
*RAD51C*	NM_058216.2	*	**									Fanconi anemia (FA-O) (recessive)
*RAD51D*	NM_002878.3	*	**									Fanconi anemia (FA) (recessive)
Low risk/insufficient data (*)												
*BARD1*	NM_000465.2	*	*									
*BLM*	NM_000057.2	*		*								Bloom Syndrome (BS)
*CHEK1*	NM_001114121.2	*										
*ABRAXAS1 (FAM175A)*	NM_139076.2	*										
*MRE11 (MRE11A)*	NM_005591.3	*										Ataxia-Telangiectasia-like disorder
*NF1*	NM_000267.3	*								*	*	Neurofibromatosis type 1
*RAD50*	NM_005732.3	*										Nijmegen breakage syndrome-like disorder (NBSLD)
*RAD51B*	NM_133509.3	*										
*XRCC2*	NM_005431.1	*										

^a^ *BRCA1, BRCA2, CDH1, EPCAM, MEN1, MLH1, MSH2, MSH6, MUTYH, PALB2, PMS2, PTEN, STK11, TP53, ATM, BRIP1, CHEK2, NBN, RAD51C, RAD51D, BARD1, BLM, ABRAXAS1, MRE11, RAD50, XRCC2* were included in the first version of the HerediGENE panel (26 gene panel) whereas *APC, BMPR1A, BRCA1, BRCA2, CDH1, CDK4, CDKN2A, EPCAM, MEN1, MLH1, MSH2, MSH6, MUTYH, PALB2, PMS2, PTEN, RET, SMAD4, STK11, TP53, VHL, ATM, BRIP1, CHEK2, NBN, RAD51C, RAD51D, BARD1, CHEK1, MRE11, NF1, RAD50, RAD51B* were included in the second version of the HerediGENE panel (33 gene panel)

### DNA isolation

Genomic DNA was extracted from peripheral blood leukocytes using the QIAamp DNA Blood Mini Kit (QIAGEN) or MagCore® Genomic DNA Whole Blood Kit (RBC Bioscience) and was quantified using NanoDrop 2000c Spectrophotometer (Thermo Fisher Scientific).

### Library preparation for NGS analysis

The analysis of genes involved in hereditary cancer predisposition was performed using two different library reparation approaches. The first 451 individuals were analyzed using an amplicon-based method, while the following 746 individuals were analyzed using a solution-based capture approach.

#### Amplicon-based gene panel protocol

Amplification of the entire coding region including the intron-exon boundaries of 26 genes (Table 1) was carried out using the RUO BRCA Hereditary Cancer MASTR™ Plus assay kit (Multiplicom NV, Agilent) according to the manufacturer’s instructions [11]. Briefly, the assay generated a library of 561 gene-specific amplicons in two rounds of PCR: Initially, for each sample, 50 ng of DNA was used to perform 5 multiplex PCR reactions which amplified the entire target region. The products were then pooled for each DNA sample and small residual DNA fragments were removed by use of a magnetic bead-based DNA purification approach. The products for each sample were used as template for a Universal PCR reaction using hybrid primers to unambiguously tag each amplicon with a unique multiplex identifier (MID) and a platform specific primer. Finally, the purification of each tagged amplicon library was performed using Agencourt AMPure XP beads (Beckman Coulter, Brea, CA, USA). Each library was quantified using NanoDrop 2000c Spectrophotometer (Thermo Fisher Scientific) to allow for the equimolar pooling of all sample libraries for subsequent sequencing.

#### Solution-based capture protocol

A probe library (Roche NimbleGen SeqCap EZ Choice) targeting all coding exons and 50 bp of flanking intronic regions of 33 genes associated with inherited cancer predisposition (Table 1) was custom designed. The sample preparation was performed according to the SeqCap EZ Choice Library User’s Guide (Roche NimbleGen). Briefly, the assay generates a library based on a solution-based capture method that enables enrichment of targeted regions from genomic DNA. Initially, for each sample, 100 ng – 500 ng of double-stranded DNA was used for enzymatic fragmentation (Kappa Hyperplus kit). EDTA neutralizing conditioning solution was used prior to fragmentation in order to ensure the stability of the enzymatic fragmentation reaction. The fragmented DNA samples were then subjected to end-repair, A-tailing and ligation of paired-end indexed adapters. Finally, the library was amplified by ligation-mediated PCR (four cycles) and allowed to hybridize overnight to the custom probes. Library preparation was completed by Post –Capture LM- PCR (14 cycles), according to the manufacturer’s protocol following the SeqCap EZ library preparation guide (Roche NimbleGen). The final library was quantified using the KAPA Library Quantification Kit for Next-Generation Sequencing on a Rotor-Gene 6000 system (Corbett Research, QIAGEN, Hilden, Germany).

### Sequencing

Irrespective of the library preparation approach, products were subsequently analyzed by Next Generation Sequencing (NGS) using the Illumina platform, MiSeq. Briefly, NGS was performed using the MiSeq Reagent Kit v3 (600-cycle) (Illumina, San Diego, California, United States). Indexed DNA library concentrations were quantified as described above and normalized to 4 nM. The library was denatured using 5 μl of 4 nM library and 5 μl 0.2 N NaOH. The library was diluted using Pre-chilled HT1 buffer at a final concentration 10 pM. Finally, the 10 pM library was spiked in 6% of PhiX Control v3 (Illumina, San Diego, California, United States), which provides a quality control for cluster generation, sequencing, and alignment.

Alignment to the reference sequence (hg19), variant calling and interpretation were performed in the context of clinically relevant transcripts (listed in Table 1) using the optimized algorithms included in the SeqNext module of the commercial SeqPilot suite (JSI medical systems GmbH, Germany). For mapping an enhanced BWA algorithm is utilized by SeqNext. Only basecalls with quality score of 20 or above were considered for further processing. The Regions Of Interest (ROIs) were defined as exons ±50-bp intronic sequence for all genes included in the gene panels. An automatic search for homologous regions in the genome was performed for all ROIs to exclude “background reads”. Reads that match to active homologous sequences were filtered and not aligned to ROIs. Any potential target region or variant position with coverage of fewer than 50 reads was reviewed by analysts and analyzed by capillary sequencing if suspect. Target regions showed an average read coverage of 900x with a minimum depth of >50x for 99% of bases. Variants were called with a variant allele frequency (VAF) cutoff of 20% and each assessed for pathogenicity as described in the Variant classification and Bioinformatics analysis section.

### DNA sanger sequencing

All pathogenic, likely pathogenic variants and VUS were confirmed by Sanger Sequencing by performing a new DNA preparation from an alternative blood vial obtained from the tested individual (primer sequences and conditions available upon request). PCR products’ purification was performed using NucleoFast® 96 PCR Clean-up kit (Macherey-Nagel GmbH and Co., Düren, Germany), according to the manufacturer’s instructions. The sequencing reactions were carried out from 2 μl purified PCR product using the BigDye® Terminator v1.1 Cycle Sequencing kit (Applied Biosystems, Foster City, CA, USA). Sequencing reaction products were purified prior to electrophoresis using the Montage™ SEQ96 Sequencing Reaction kit (EMD Millipore Corp., Billerica, MA, USA). Electrophoresis of sequencing products was conducted on an Applied Biosystems 3130 Genetic Analyzer (Applied Biosystems).

### Large genomic rearrangement (LGR)

Analysis of Large Genomic Rearrangements (LGR) for genes in which such mutational events have been previously described was carried out. Specifically, the following genes were analyzed in both panels: *BRCA1, BRCA2, CHEK2, EPCAM (*Exons 8, 9*), MLH1, MSH2, MSH6, MUTYH, PALB2, RAD50 (*Exons 1, 2, 4, 10, 14, 21, 23 and 25*), RAD51C, RAD51D,* and *TP53.*

For this purpose, Multiplex Ligation-dependent Probe Amplification (MLPA) analysis was performed for every sample analyzed by the amplicon-based method using the appropriate MLPA probe mix and according to manufacturer’s instructions: *BRCA1*: P002; *BRCA2*: P045; *CHEK2*: P190; *EPCAM*, *MSH6*; P072, *MLH1*, *MSH2*; P003; *MUTYH*: P378; *PALB2*, *RAD50*, *RAD51C*, *RAD51D*: P260; *TP53*: P056 (MRC Holland). An Applied Biosystems 3130 Genetic Analyzer was used for electrophoresis and the Coffalyser.Net software was used for the analysis.

In contrast to amplicon-based methods, capture-based approaches provide better uniformity of coverage [12]. Thus the capture-based approach allowed for computational analysis of LGRs from NGS data. For this purpose the CNV module of the software suite SeqPilot (JSI Medical Systems) and panelcn.MOPS [13] were used. Both algorithms are specifically developed for CNV analysis of sequencing data reporting 99–100% sensitivity and up to 100% specificity for the prediction of Large Genomic Rearrangements up to the level of a single gene exon. All LGRs detected with these algorithms were then verified experimentally using the MLPA technique as described above.

### RNA extraction and RT-PCR

Peripheral blood mononuclear cells were separated from the other components of the blood by gradient centrifugation using Ficoll-Paque™ PLUS Media (Fischer Scientific). Total RNA was then extracted using Trizol reagent (Invitrogen, Paisley, UK), following standard protocol provided by the manufacturer. cDNA was synthesized using SuperScript™ VILO™ cDNA Synthesis Kit (Thermo Fisher Scientific) as described by the supplier. Primer sets used for PCR amplification are available upon request.

### Variant classification and bioinformatics analysis

The annotation and interpretation of all identified variants was performed using an in-house local knowledge-base and a proprietary bioinformatics pipeline designed for the automation of the classification process. The clinical significance of all identified variants was examined using the standards and guidelines for the interpretation of sequence variants recommended by the American College of Medical Genetics and Genomics (ACMG Laboratory Quality Assurance Committee) and the Association for Molecular Pathology (AMP) [14]. Minor Allele Frequencies were examined through access to population databases and in specific to the Genome Aggregation Database (gnomAD) [15], the Exome Aggregation Consortium (ExAC) [15], the 1000 Genomes Project [16], the Kaviar [17], the NHLBI Exome Sequencing Project ESP6500 [18] and the Greater Middle East (GME) Variome Project [19] databases. Disease specific information for variants were retrieved from ClinVar [20], OMIM [21], and the Leiden Open Variation Database (LOVD) [22]. The impact of missense substitutions on protein structure and function was analyzed using the consensus predictive (in silico) algorithm MetaSVM [23] and Align GVGD (Grantham Variation, Grantham Deviation) [24]. The nucleotide conservation of all variants was examined through phyloP [25] and SiPhy [26]. Protein features and domain specific information were retrieved from the UniProt database [27]. The effect of variants on splicing was in silico examined using Human Splicing Finder [28]. All variant information and lines of evidence used for classification were stored, organized and continuously updated and upgraded in an in-house local knowledge-base. This also enabled a reproducible, rigorous and efficient reclassification process.

### Statistical analyses

Pearson correlation analysis with ‘N-1’ correction ([29, 30]) was performed for the correlation between two parameters. Differences in the distribution of continuous variables between categories were analyzed by Mann–Whitney U test. All analyses were performed with R software version 3.4.4 ([31]). All statistical tests were 2-sided, and an adjusted *P* < 0.05 was considered statistically significant.

## Results

### Patient demographics

During the time period between June 2014 and February 2018, 1197 individuals were referred to our laboratory for genetic testing and specifically 631 individuals from Greece (52.7%), 408 from Romania (34.1%) and 158 from Turkey (13.2%). The median age at testing of individuals in our cohort was 45 years old (range 8 months – 87 years old). The majority of individuals tested were female (94%, 1126/1197) while only 6% (71/1197) were male.

Among the 1197 cases referred for testing, 77.6% (929/1197) had a personal history of cancer, 11.8% (141/1197) were unaffected at the time of testing, while for 10.6% (127/1197) of individuals no clinical data was available. The median time from diagnosis to testing was 1 year with approximately 73% (678/929) of affected individuals tested within 12 months of diagnosis. The majority (82.7%) (768/929) of affected individuals had a personal history of breast cancer. A family history of cancer constituted a major reason for referral, accounting for 84.0% (780/929) and 91.5% (129/141) of affected and unaffected individuals respectively. Among unaffected individuals with a family history of cancer, approximately 87% (112/129) had at least one first−/second-degree relative with a median of 3 relatives with history of any cancer. The subgroup of affected individuals with other cancers included patients with personal history of the following cancer types/sites reported: abdomen, adenocarcinoma, bile, brain, endocrine glands, endometrium, fallopian tubes, gastric, gynecological, Hodgkin’s, kidney, lymphoma, leukemia, liver, melanoma, pancreas, peritoneum, prostate, sarcoma, stomach, thyroid, unknown origin, uterine fibroma.

Individuals tested from Greece had the highest number of affected individuals (81.3%; 513/631) compared to individuals from Turkey (74.7%; 118/158) and Romania (73.0%; 298/408). In addition, among affected individuals from Greece, 87.3% (448/513) reported family history of cancer whereas in Turkey and Romania, 84.7% (100/118) and 78.2% (233/298) of affected individuals reported family history of cancer respectively. These differences underline the different criteria that the ordering physicians used for the selection of patients for genetic testing and the heterogeneous background of these populations. The detailed demographic and clinical features of the individuals are summarized in Table 2.

**Table 2:** Demographic and clinical characteristics for individuals tested with the hereditary cancer panel

Demographic	No.	%
Total individuals	1197	100
Female	1126	94.1
Male	71	5.9
Age at diagnosis (years)		
Mean ± SD	45.4 ± 11.4	
Median	44	
Range	0.7–86	
Age at testing (years)		
Mean ± SD	46.1 ± 11.5	
Median	45	
Range	0.7–87	
Ethnicity		
Greek	631	52.7
Romanian	408	34.1
Turkish	158	13.2
Clinical status		
Affected	929	77.6
Unaffected	141	11.8
No information	127	10.6
Cancers among affected patients		
Breast	768	82.7
Ovarian	41	4.4
Colorectal	68	7.3
Other	61	6.6
Family history of unaffected individuals		
Breast cancer	103	73.0
Ovarian cancer	30	21.3
Colorectal cancer	32	22.7
Prostate cancer	22	15.6
Pancreatic cancer	16	11.3
Breast cancer & Ovarian cancer	19	13.5
Breast cancer & Colorectal cancer	18	12.8
No cancer	6	4.3
Unknown	3	2.1

### *MUTYH* variants

In the *MUTYH* gene, five variants c.536A > G p.(Tyr179Cys), c.734G > A p.(Arg245His), c.884C > T p.(Pro295Leu), c.1187G > A p.(Gly396Asp), c.1437_1439delGGA p.(Glu479_Glu480delinsGlu) accounted for all 24 *MUTYH* pathogenic cases identified in our cohort. In only 3 cases the *MUTYH* alterations were present in homozygosis or compound heterozygosis. In all three cases the patient had been referred for testing due to a relevant colorectal cancer (CRC) phenotype. One case was a 44-year old male with colorectal cancer and no other history of cancer in the family. The second case was a 43-year old male with colorectal cancer and no family history of cancer. The third case was a female patient diagnosed with small intestine cancer and polyps at ages 65 and 79 with her 75 year old brother having the same clinical features.

Nine of the individuals with a monoallelic pathogenic *MUTYH* variant, also carried pathogenic variants in another gene, more relevant to the reported phenotype, with the exception of a female individual with a diagnosis of CRC at age 61 in whom the second pathogenic variant was identified in the *RET* gene, without any thyroid cancer diagnosis reported in the family. Finally, a single *MUTYH* pathogenic variant was identified in 12 individuals. Two of the individuals were unaffected at the time of testing and had no reported CRC diagnosis in the family, while 7 of the patients were tested because of a breast cancer diagnosis and only one of them had a CRC diagnosis in the family (her father and his two sisters). The remaining 3 individuals in whom a single pathogenic *MUTYH* variant was identified were diagnosed with CRC at ages 61 (no other CRC in the family) and 51 (one SDR with CRC at 67 years), and FAP phenotype at age 45 with a strong family history of FAP phenotype.

### Pathogenic and likely pathogenic findings

**Fig. 1:**
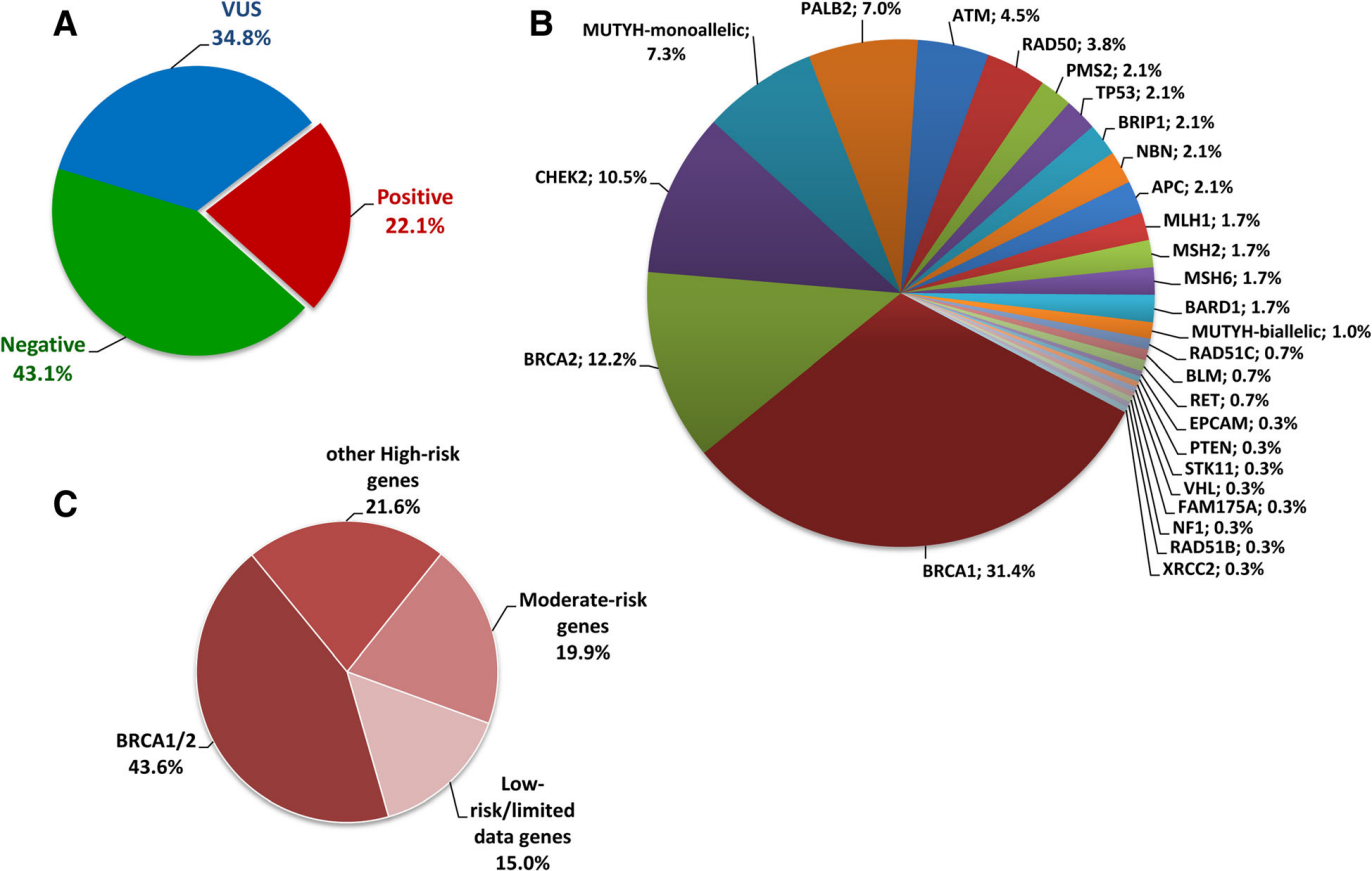
Panel testing outcomes and positive results for the 1197 individuals tested grouped by gene and gene category based on risk for any cancer type (Table 1). **a.** Outcomes of panel testing for the 1197 individuals tested. Positive results refer to the cases where a pathogenic/likely pathogenic variant was identified **b.** Percentage of pathogenic/likely pathogenic findings identified in each gene **c.** Pathogenic/likely pathogenic findings stratified by gene risk category for any cancer type

DNA from a total of 1197 individuals was analyzed by one of the two panels described in Table 1. The first 451 individuals were analyzed using the 26 gene panel, while the following 746 individuals were analyzed using the 33 gene panel. Both panels included genes associated with high, intermediate and low cancer risk. At least one clinically significant variant was identified in 264 of samples (22.1%) (Fig. 1a) including individuals with no available information about their personal or family history of cancer. The mutation frequency among the individuals of Greek, Romanian and Turkish ethnicity was 20.4% (129/631), 27.0% (110/408) and 15.8% (25/158), respectively (Table 3). Clinically significant alterations were identified in 27 of the 36 genes analyzed (See Additional file 2: Table S1), with a total of 161 unique pathogenic and likely pathogenic variants being detected among the 264 carriers (See Additional file 3 Table S2, Additional file 4: Table S3, and Fig. 1b). Notably, 28 of the 161 unique variants identified in our cohort had never been reported before in variant databases [20]. Frameshift mutations were the most prevalent mutation type, accounting for the 34.2% (55/161) of the variants identified, followed by nonsense, missense, splicing mutations, large rearrangements and in frame insertion/deletion mutations (26.7% (43/161), 16.8% (27/161), 14.3% (23/161), 6.8% (11/161) and 1.2% (2/161) respectively) (See Additional file 1: Figure S1A).

**Table 3:** Frequency of Pathogenic and Likely Pathogenic variants among tested individuals

Individuals	Positive yield % (positive individuals/total individuals)	Positive yield in gene categories % (positive individuals/total individuals)			
		*BRCA1* and *BRCA2*	Other high-risk genes	Genes with moderate risk	Genes with low/unknown risk
Total individuals	22.1% (264/1197)	10.5% (126/1197)	5.2% (62/1197)	4.8% (58/1197)	3.3% (44/1197)
Greek	20.4% (129/631)	8.2% (52/631)	5.4% (34/631)	5.2% (33/631)	3.8% (24/631)
Romanian	27.0% (110/408)	14.2% (58/408)	5.4% (22/408)	5.9% (24/408)	3.7% (15/408)
Turkish	15.8% (25/158)	10.1% (16/158)	4.4% (7/158)	0.6% (1/158)	1.9% (3/158)
Affected individuals	24.2% (225/929)	11.2% (104/929)	5.9% (55/929)	5.8% (54/929)	3.9% (36/929)
Breast cancer	24.7% (190/768)	12.6% (97/768)	3.9% (30/768)	6.4% (49/768)	3.5% (27/768)
Colorectal cancer	27.9% (19/68)	2.9% (2/68)	25.0% (17/68)	2.9% (2/68)	7.4% (5/68)
Ovarian cancer	19.0% (8/42)	14.3% (6/42)	2.4% (1/42)	2.4% (1/42)	0.0% (0/42)
Other cancers	21.3% (13/61)	4.9% (3/61)	8.2% (5/61)	3.3% (2/61)	3.3% (2/61)
Unaffected individuals	14.9% (21/141)	7.1% (10/141)	3.5% (5/141)	2.8% (4/141)	2.1% (3/141)
FH of Breast cancer	14.6% (15/103)	8.7% (9/103)	1.0% (1/103)	1.9% (2/103)	2.9% (3/103)
FH of Colorectal cancer	21.9% (7/32)	6.3% (2/32)	9.4% (3/32)	6.3% (2/32)	0.0% (0/32)
FH of Ovarian cancer	23.3% (7/30)	13.3% (4/30)	0.0% (0/30)	10.0% (3/30)	3.3% (1/30)
Individuals with no information	14.2% (18/127)	9.4% (12/127)	3.1% (4/127)	0.8% (1/127)	0.8% (1/127)

The most prevalent variant was the frameshift c.5266dupC p.(Gln1756Profs*74) in the *BRCA1* gene which was detected in 26 probands in all three nationalities tested (See Additional file 3: Table S2). The second most common *BRCA1* variant was the nonsense mutation c.3607C > T p.(Arg1203*), which was absent in the Greek population tested, but was detected in 9 Romanians and 1 individual of Turkish origin. Additionally, the low penetrance missense *CHEK2* variant c.470 T > C p.(Ile157Thr) was identified in 16 cases, which is the second most common variant detected. This variant has been shown to increase the risk of breast and colorectal cancer [32–34].

### Large genomic rearrangements (LGRs)

Of notice is also the relatively high percentage of large genomic rearrangements identified (See Additional file 4: Table S3). Of the 16 LGRs detected, 10 occurred in the *BRCA1/2* genes, 2 in *MSH2*, 2 in *CHEK2*, while a single LGR was detected in *EPCAM, MLH1* and *PMS2*.

### Pathogenic variants in high-, moderate- and low-risk genes

Among the individuals with a pathogenic finding, 43.6% (126/289) of the alterations identified cases, the alteration identified occurred in the *BRCA1* or *BRCA2* genes, indicating the significant contribution of these two genes in hereditary cancer predisposition. The *BRCA1* gene was found to be mutated in 90 individuals (with a mutation frequency of 7.5% (90/1197) among the entire cohort), while *BRCA2* was mutated in 36 cases (percentage of 3.0% (36/1197) of the cases tested). This finding was expected given the prevalence of breast and ovarian cancer personal and family history in our cohort. Concerning the mutation distribution among individuals with positive findings, 56.4% of the alterations detected were located in one of the other hereditary cancer related genes (21.6, 19.9, and 15.0% in high, moderate and low risk genes respectively) (Fig. 1c). If molecular analysis was restricted to *BRCA1/2* only the hereditary etiology of cancer would have been identified in 10.5% (126/264) of the cases. The analysis of the other high penetrance genes of the panel increases the percentage of alterations detected by 4.3%, while the analysis of the moderate/low penetrance genes leads to the identification of an additional 7.4% pathogenic variants (4.1 and 3.3% for moderate and low penetrance genes, respectively) (Table 3). Apart from the *BRCA1/2* genes other highly mutated genes are *CHEK2* (2.5%), *MUTYH* (1.8% monoallelic variants, 0.3% biallelic variants), *PALB2* (1.7%), *ATM* (1.2%) and *RAD50* (0.9%).

Among individuals with personal history of breast and/or ovarian cancer we observed a statistically significant difference in the mutation frequencies between high risk and moderate risk genes (16.5% versus 6.2%, *p* < 0.0001). There was also significant difference between the positive rates in high risk of medium risk genes based on the affection status, especially for breast cancer. For example, we observed a significantly increased mutation rate in high risk genes in individuals with personal history of breast/ovarian cancer compared to unaffected individuals with family history of breast/ovarian cancer (16.5% versus 8.8%, *p* = 0.0338).

### Pathogenic variants per cancer type

Considering the type of gene altered in relation to the cancer type or the cancer family history of the patient, we observed a high prevalence of *BRCA1* and *BRCA2* pathogenic variants in both patients with personal and family history of breast cancer (12.6% (97/768) and 8.7% (9/103), respectively). A pathogenic variant in other high penetrance genes was detected in 3.9% (30/768) of breast cancer patients and in 1.0% (1/103) of the patients with breast cancer family history, while the moderate/low penetrance gene mutation rate was 9.9% (76/768) and 4.8% (5/103), respectively. In total, at least one pathogenic variant was detected in 24.7% (190/768) of breast cancer affected individuals and 14.6% (15/103) of those with family history of breast cancer. Among individuals with positive findings and a personal history of breast cancer, the mutation distribution for high, moderate and low risk genes was 62.1, 24.1 and 13.8%, respectively. In addition to *BRCA1/2* other highly mutated genes in those patients were *PALB2, CHEK2, MUTYH* and *ATM* (See Additional file 1: Figure S2).

Among patients with colorectal cancer, 27.9% (19/68) of the affected patients and 21.9% (7/32) of the individuals with colorectal cancer family history presented a pathogenic variant in at least one of the genes tested. The presence of a pathogenic variant in the MMR genes *MLH1*, *MSH2* and *MSH6* was the most common finding in these patients followed by monoallelic alterations in *MUTYH* and *APC* alterations. In the limited number of individuals tested because of a personal or family history of ovarian cancer (42 and 30 cases, respectively), a pathogenic/likely pathogenic variant was identified in 19.0% (8/42) and 23.3% (7/30) of cases. In patients with a personal history of cancer other than breast, ovarian and colorectal, a pathogenic/likely pathogenic variant was identified in 21.3% (13/61) of the cases. In this group of individuals the percentage of *BRCA1/2* mutated cases was as expected lower (4.9% (3/61)). Finally, in tested individuals with no information about personal/family history of cancer, a clinically significant variant was identified in 14.2% (18/127) of the cases with 9.4% (12/127) in *BRCA1/2* genes and 4.7% (6/127) in other high, moderate and low risk genes (Table 3).

### mRNA splicing variants

Splicing variants are considered to be a common cause of cancer susceptibility. A total of 23 splicing variants were identified at 5’or 3’of the exon and one variant occurred at the last exonic nucleotide. Of those 19 were already described in international bibliography or in variation databases and were classified as pathogenic. For the remaining 5 cases, mRNA was requested for better classification of the splicing variant. In three cases mRNA was not available, while for two individuals, carrying splicing variants in *PALB2* and *BMPR1A*, mRNA analysis was carried out.

**Fig. 2:**
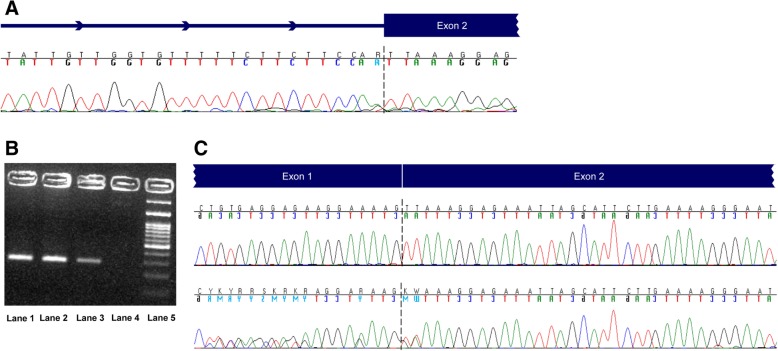
mRNA analysis of the c.49-1G > A variant in *PALB2* **a.** Chromatograms of sequencing analysis of genomic DNA of a patient carrying the c.49-1G > A variant in *PALB2*. **b.** RT-PCR products on 3% agarose gel. Lanes 1 and 2: the sample of the patient with the variant, Lane 3: normal sample, Lane 4: negative control, Lane 5: 100 bp DNA Ladder (New England Biolabs). **c.** Chromatograms of sequencing analysis of cDNA from the same patient showing that this splicing variant leads to the in-frame deletion of two amino acid residues, p.Leu17_Lys18 (bottom panel) compared to the sequencing analysis of a wild type sample (top panel)

The first variant was *PALB2* c.49-1G > A, which was identified in a 54 year-old female, who was affected by breast cancer at the age of 49, with a family history of diverse cancers. This alteration was a replacement of the last nucleotide base of intron 1 of the *PALB2* gene. Since this particular location is strictly conserved in human and other genomes, it was expected that incorrect mRNA splicing occurred with subsequent production of a truncated and non-functional protein. This alteration had not been described in variant databases. We therefore undertook mRNA analysis in order to better classify it by determining its impact at the RNA level. This analysis indicated that the variant leads to the elimination of two amino acid residues p.(Leu17_p.Lys18) (Fig. 2), which are known to be in a functionally relevant region of the protein [35–37]. Specifically, a conserved coiled-coil motif is present at the N-terminus of the PALB2 protein (aa 9–42) and mediates the PALB2-BRCA1 protein-protein interaction through a similar motif in the BRCA1 protein (aa 1393–1424) [35–37]. This coiled-coil domain has also been reported to mediate PALB2 dimerization or oligomerization [38, 39] suggesting a possible competition between the PALB2-PALB2 self-interaction and the PALB2-BRCA1 complex formation. Using site directed mutagenesis several residues within the coiled-coil motif have been shown as important for the hetero-oligomeric interaction between PALB2 and BRCA1 as well as the PALB2 dimerization or oligomerization leading to reduced HR activity [40]. Interestingly the p.Lys18Ala variant of PALB2, although not affecting the BRCA1-PALB2 complex formation, exhibited reduced PALB2 HR activity, suggesting that the variant may affect the integrity of the coiled-coil motif and that even a modest distortion of the structure could result in reduced HR activity, even if the binding of BRCA1 is not affected [40].

**Fig. 3:**
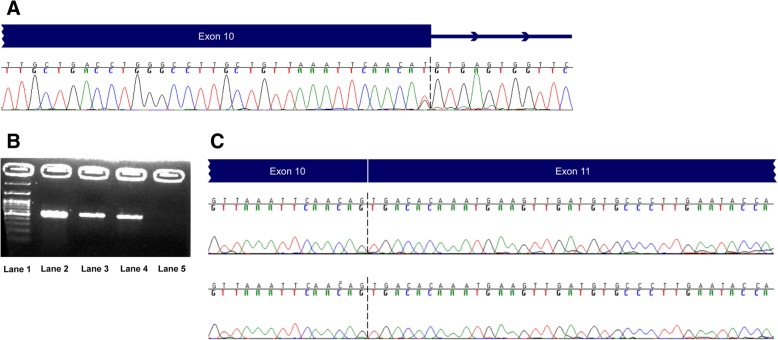
mRNA analysis of the c.1166G > T in *BMPR1A* **a.** Chromatograms of sequencing analysis of genomic DNA of a patient carrying the c.1166G > T in *BMPR1A*. **b.** RT-PCR products on 3% agarose gel. Lane 1: 100 bp DNA Ladder (New England Biolabs), Lanes 2 and 3: the sample of the patient with the variant, Lane 4: normal sample, Lane 5: negative control. **c.** Chromatograms of sequencing analysis of cDNA A from the same patient. The c.1166 T variant is not present, indicating instability of the aberrantly spliced transcript (top panel: wild type sample, bottom panel: sample of the patient with the variant)

The second case was a 38 year-old breast cancer female who presented the c.1166G > T, p.(Ser389Ile) in the *BMPR1A* gene. This alteration was located in the last nucleotide of exon 10, a strictly conserved region in human and other genomes. Algorithms developed to predict the effect of single nucleotide changes on mRNA processing, predicted that this change may alter splicing of the resultant mRNA but this prediction had not been confirmed experimentally, thus an RNA analysis of this area was undertaken. The RT-PCR analysis did not reveal any change in the length of the RNA produced (Fig. 3). However, Sanger sequencing analysis revealed the absence of the altered nucleotide c.1166 T at the RNA level, with only the wild type allele c.1166G detected. The most probable explanation for this finding is that the altered c.1166 T allele produces an incorrectly spliced and unstable transcript. Even if this finding is an indication of the pathogenicity of this variant, additional analysis at the protein level is required in order to classify it as pathogenic.

### Multiple pathogenic variants

Two cancer predisposition causing variants were identified in 25 individuals (Additional file 5: Table S4). In the majority of the cases (15 individuals), a personal history of breast cancer was reported. In 2 cases both pathogenic variants were located in high penetrance genes, while in the remaining cases at least one of the genes mutated belonged to the moderate or low penetrance group. Furthermore, in two cases the individuals carried two pathogenic variants in the same gene (*MUTYH* and *CHEK2* respectively). The most common alteration identified in this group was the low penetrance *CHEK2* variant p.(Ile157Thr), which was detected in 4 cases.

### Variants of uncertain significance (VUS)

The overall VUS rate among the 1197 individuals in our cohort was 34.8% (417/1197) with a maximum of 4 VUS per individual (Table 4). In particular, 72.4% (302/417) of individuals had 1 VUS detected whereas in 23.5% (98/417), 3.1% (13/417) and 1.0% (4/417) of individuals, 2, 3 or 4 VUS were identified, respectively (See Additional file 1: Figure S4A). There was a statistically significant difference (*p* = 0.0122) in the VUS rate between individuals from Southeastern Europe (Greece and Romania) and Western Asia (Turkey) with 33.5% (348/1039) of individuals from the Balkan Peninsula receiving a VUS as a result compared to 43.7% (69/158) of individuals tested from Turkey. Similar VUS rates were observed among affected and unaffected individuals and their subcategories (Table 4). The highest VUS rate was observed among unaffected individuals with family history of Breast cancer (41.7% (43/103)) and among individuals with personal history of cancer other than Breast, Ovarian or Colorectal (41.0% (25/61)) cancer. The lowest percentages of VUS were observed in individuals with personal history of Ovarian cancer (31.0% (13/42)) and among unaffected individuals with family history of colorectal cancer (31.3% (10/32)). At least one VUS was identified in all genes tested by the two versions of the hereditary cancer panel (See Additional file 6: Table S5), with most variants detected in the *ATM* gene where 59 unique variants were found in 88 individuals (7.3%). Approximately, 43.0% of VUS were detected in high-risk genes whereas 31.9 and 25.1% were detected in moderate- and low-risk genes respectively (See Additional file 1: Figure S4B and S4C). The vast majority of the 424 unique variants of uncertain significance detected were missense mutations (See Additional file 1: Figure S1B) resulting in conservative (54.5% (231/424)), non-conservative (22.4% (95/424)) and radical (23.1% (98/424)) amino acid substitutions predicted at the protein level (See Additional file 6: Table S5).

**Table 4:** Frequency of Variants of Uncertain Significance (VUS) among tested individuals

Individuals	% VUS rate	% VUS rate			
		*BRCA1* and *BRCA2*	Other high-risk genes	Genes with moderate risk	Genes with low/unknown risk
Total individuals	34.8% (417/1197)	3.0% (36/1197)	15.5% (185/1197)	13.0% (156/1197)	10.1% (121/1197)
Greek	35.5% (224/631)	2.2% (14/631)	16.2% (102/631)	13.8% (87/631)	10.9% (69/631)
Romanian	30.4% (124/408)	2.5% (10/408)	13.2% (54/408)	11.3% (46/408)	9.3% (38/408)
Turkish	43.7% (69/158)	7.6% (12/158)	18.4% (29/158)	14.6% (23/158)	8.9% (14/158)
Affected individuals	34.8% (323/929)	2.8% (26/929)	15.6% (145/929)	12.3% (114/929)	10.3% (96/929)
Breast cancer	34.9% (268/768)	2.6% (20/768)	15.2% (117/768)	10.7% (82/768)	6.4% (49/768)
Colorectal cancer	36.8% (25/68)	2.9% (2/68)	13.2% (9/68)	8.8% (6/68)	11.8% (8/68)
Ovarian cancer	31.0% (13/42)	4.8% (2/42)	16.7% (7/42)	4.8% (2/42)	4.8% (2/42)
Other cancers	41.0% (25/61)	3.3% (2/61)	24.6% (15/61)	8.2% (5/61)	4.9% (3/61)
Unaffected individuals	39.0% (55/141)	2.8% (4/141)	16.3% (23/141)	19.1% (27/141)	9.2% (13/141)
FH of Breast cancer	41.7% (43/103)	2.9% (3/103)	15.5% (16/103)	22.3% (23/103)	9.7% (10/103)
FH of Colorectal cancer	31.3% (10/32)	3.1% (1/32)	15.6% (5/32)	12.5% (4/32)	6.3% (2/32)
FH of Ovarian cancer	36.7% (11/30)	0.0% (0/30)	10.0% (3/30)	20.0% (6/30)	10.0% (3/30)
Individuals with no information	30.7% (39/127)	4.7% (6/127)	13.4% (17/127)	11.8% (15/127)	13.4% (17/127)

In the group of individuals with a personal history of breast cancer, 34.9% (268/768) received at least one VUS in their report with 2.6% (20/768) having a VUS in the *BRCA1/2* genes, 15.2% (117/768) in other high-risk genes for breast cancer (*CDH1, PTEN, STK11, TP53,* and *PALB2*), 10.7% (82/768) in moderate-risk genes for breast cancer (*ATM, CHEK2* and *NBN*) and 6.4% (49/768) in low-risk genes or with limited information. The gene with the highest VUS rate among the entire cohort was *ATM* (7.4% (88/1197)), followed by *RAD50* (2.8% (34/1197)), *CHEK2* (2.4% (29/1197)) and *APC* (2.0% (24/1197)). Among high-risk genes for breast cancer *BRCA2* (1.9% (23/1197)) and *PALB2* (1.7% (20/1197)) had the highest VUS rates. *ATM*, *APC* and *BRCA2* have the largest total coding region and could therefore be susceptible to more variation as observed before [41].

Approximately 19 variants (4.5% of VUS reported) have been reclassified since they were reported (affecting 7.7% (32/417) of individuals with a VUS). Variant reclassification in our cohort resulted in a 2.5% decrease of the VUS rate (See Additional file 1: Figure S5). All revised reports included a variant being downgraded from uncertain significance to likely benign classification mostly because of increasing observations in the tested population and/or new information from variant databases or large datasets.

## Discussion

In the present study multi gene variant analysis was applied using NGS technology for the detection of hereditary cancer related pathogenic mutations. In total, 1197 consecutive individuals referred to our laboratory for analysis of hereditary cancer predisposition genes were included in the study. Personal and family history was available for 85.5% (1023/1197) of the individuals analyzed. In the majority of cases (78.7% (942/1197)), the reason for referral was a personal or family history of Breast and/or Ovarian cancer and affected individuals were tested within 1 year of diagnosis. This is indicative of a bigger awareness in terms of preventive diagnosis for this tumor type, especially following the publicity received by the *BRCA1* and *BRCA2* genes due to the “Jolie effect” [42, 43]. Variant analysis revealed the presence of at least one clinically significant variant in 22.1% of the individuals analyzed, while a VUS was identified in 34.8% (417/1197) of the cases. A *BRCA1* or *BRCA2* alteration was the most commonly identified finding, accounting of 47.8% of the pathogenic variants detected in our cohort. However, the contribution of other genes to hereditary cancer predisposition is also of great significance since approximately 52% of the pathogenic variants detected were located in another gene of the panel (Table 3, Fig. 1). Therefore, analysis of only *BRCA1* and *BRCA2* would explain the genetic etiology of cancer in just 10.5% (126/1197) of the individuals in our cohort. However, the analysis of the other high penetrance genes of the panel increased this percentage by 4.5%. A further increase of 7% was achieved by analyzing the moderate/low penetrance genes (Table 3). The significant contribution of additional genes, other than *BRCA1* and *BRCA2*, was also observed in breast cancer patients. In addition to *BRCA1* and *BRCA2* other highly mutated genes in those patients were *PALB2* (1.7% (13/768)), *CHEK2* (3.5% (27/768)), *MUTYH* (1.4% (11/768)) and *ATM* (1.4% (11/768)) (See Additional file 1: Figure S2). *BRCA1* and *BRCA2* pathogenic variants were identified in 12.6% (97/768) of breast cancer patients, whereas, 2.5% (19/768) of the individuals carried a variant in another high risk gene and 9.7% (74/768) in a moderate/low penetrance gene (Fig. 6).

It is evident from these findings that there is a considerable probability of identifying a pathogenic variant in a moderate/low risk gene. For some of these genes the available data concerning cancer risk and carrier’s clinical management are limited at the time being. However, the incorporation of these genes in many cancer panels and the continuous accumulation of published data from these analyses has led to the inclusion of several of them in clinical management guidelines such as those formulated by NCCN and other working groups [44–46]. Indeed, the continuous updating of the NCCN guidelines reflects the increasing understanding of further genes in Breast Cancer susceptibility and the clinical benefits of analysis of such genes. Examples of such genes are *RAD51C*, *RAD51D*, *BRIP1*, *NBN* and *NF1* while for others such as *MRE11*, *XRCC2*, *RAD51B* and *RAD50*, further studies are required to clarify the extent of their contribution in hereditary predisposition. Of note is the fact that in 88.4% of breast cancer patients who received a positive result, the variant was identified in a gene for which clinical management guidelines are available in NCCN. [44].

**Fig. 4:**
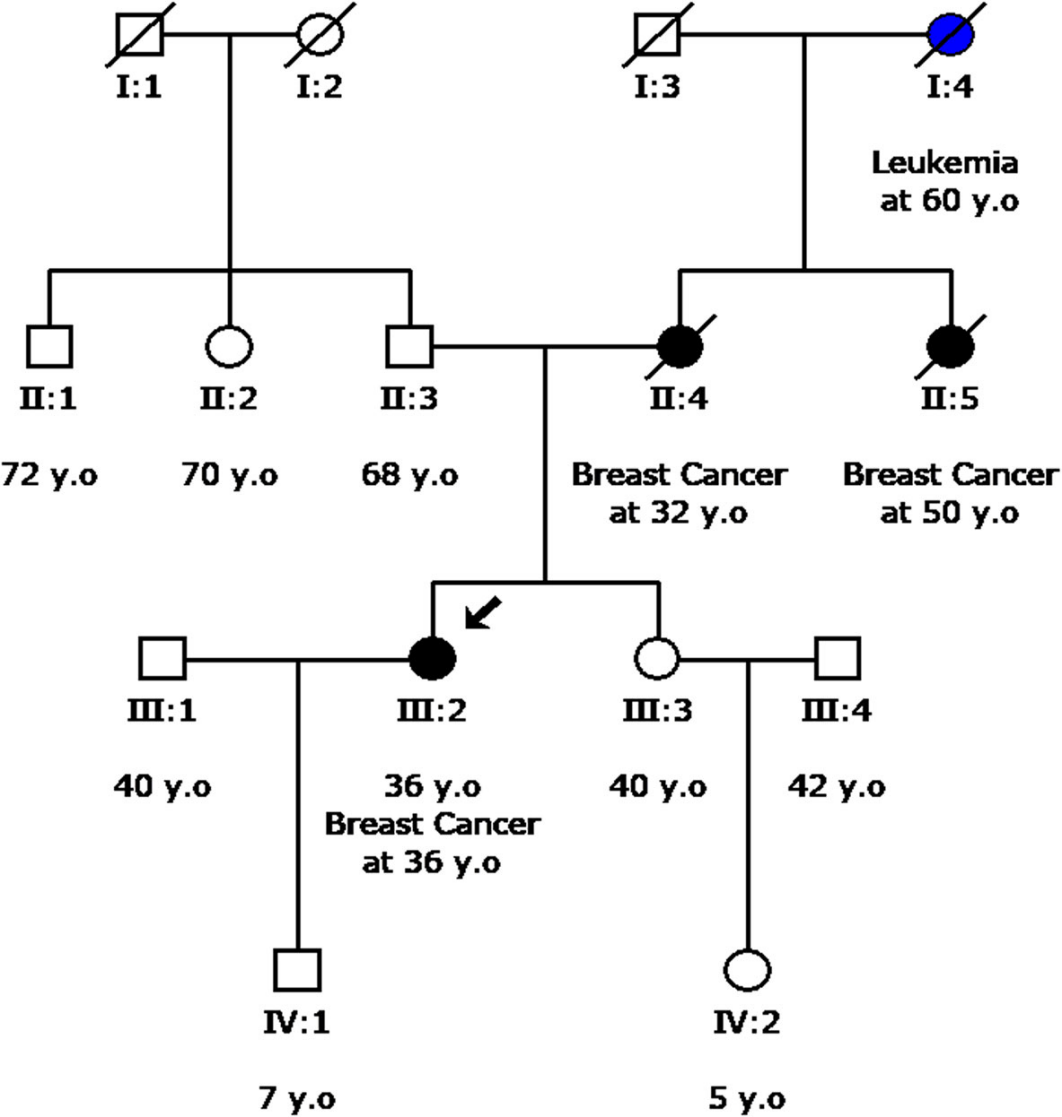
Pedigree of a family with strong breast cancer history

An example of a family which benefited from analysis of a wider panel of genes is depicted in Fig. 4. The proband in this family (III:3) was originally referred for genetic testing of *BRCA1/2* because of a strong family history of breast cancer, even though she was unaffected at the time of testing at the age of 40. No pathogenic variant was identified in the genes analyzed. However, since she was unaffected it was not clear whether the negative result was due to a variant in another gene or because she had not inherited the predisposition to breast cancer evident in her family. A year later, the sister (III:2) of the individual was referred for analysis after having been diagnosed with breast cancer at the age of 36. Multi-gene analysis revealed the presence of a pathogenic variant in the *CHEK2* gene which is associated with increased risk of breast cancer. Targeted analysis of the *CHEK2* variant in the original proband (III:3) revealed that she did not carry the variant. Thus the multi-gene analysis in this family provided superior information compared to single gene analysis for both individuals tested. The affected sister was subsequently managed clinically based on the guidelines suggested for *CHEK2* pathogenic variant carriers. The unaffected sister could forgo the increased surveillance and probably preventive surgery which would have been recommended based on her family history.

*MUTYH* was a commonly mutated gene in our cohort. Biallelic pathogenic variants in this gene are related to *MUTYH* associated polyposis (MAP) syndrome [47]. A monoallelic *MUTYH* pathogenic variant was identified in 21 individuals. Of those, only three were referred for testing due to a CRC or FAP diagnosis and only one had a family history of FAP. Limited bibliographical evidence suggests that a single *MUTYH* pathogenic variant can increase the risk of CRC by up to 2.5 times compared to the general population [48, 49]. The NCCN guidelines for Genetic/Familial High-Risk Assessment of Colorectal Cancer [45] mention that there is some evidence of a slightly increased risk of CRC in *MUTYH* heterozygotes and therefore suggest specialized screening for CRC in some carriers. In 9 of the 25 individuals harboring pathogenic variants in two genes, one of the findings was a monoallelic *MUTYH* variant. Further studies regarding the significance of monoallelic pathogenic variants in *MUTYH* are clearly needed*.*

A considerable percentage (6.8% (11/161)) of the pathogenic variants detected was LGRs. Analysis of this mutational type is essential in any comprehensive genetic testing approach. Τhis is also evident in our cohort, since 6.1% (16/264) of the individuals with positive findings had a LGR. In particular, 9% (6/65) of all *BRCA1/2* clinically significant findings identified in our cohort were LGRs. This percentage was higher in Greek patients of our cohort where 14% of *BRCA1/2* pathogenic variants were LGRs. This is in direct relation to our previous findings where 14.7% of pathogenic variants identified in *BRCA1/2* analysis were LGRs [50]. It is important to note that computational algorithms used for CNV analysis have limitations especially in terms of specificity. Therefore, the results from these algorithms should always be evaluated and confirmed by a gold standard method such as MLPA when used in the clinical diagnostics routine. We observed that the number of false positive predictions differed among genes; with more false positive duplication calls in genes/regions were high homologous pseudogenes exist.

Overall, 161 unique, clinically significant variants were identified among a total of 264 carriers. Of these, 28 had not been previously described in variant databases. Variant data sharing is crucial for the clarification of pathogenic variant frequencies, especially for genes with limited data available. In this context, all pathogenic and likely pathogenic variants identified in our study have been submitted in the ClinVar database (SUB4381212).

By using NGS technology, it has become possible to study a wider range of hereditary cancer related genes. The sequential analysis of genes has the disadvantage of been laborious, expensive and time consuming. Thus, in the past, identification of a pathogenic variant usually led to termination of the analysis and attribution of the family history in the single finding detected. However, as observed in this and previous studies [4, 51, 52] a remarkable 9.5% of individuals (25 out of 264) with pathogenic findings harbored variants in more than one genes of the panel. Each altered gene can independently increase the risk for different tumor types while their combined effect in many cases has not been studied. For example, if pathogenic variants are identified in both a breast cancer and a colorectal cancer susceptibility gene in a single individual, subsequent surveillance should take into consideration both alterations. Similarly, relatives of the patient should be screened for both variants, as both have an equal possibility of being shared by the family.

**Fig. 5:**
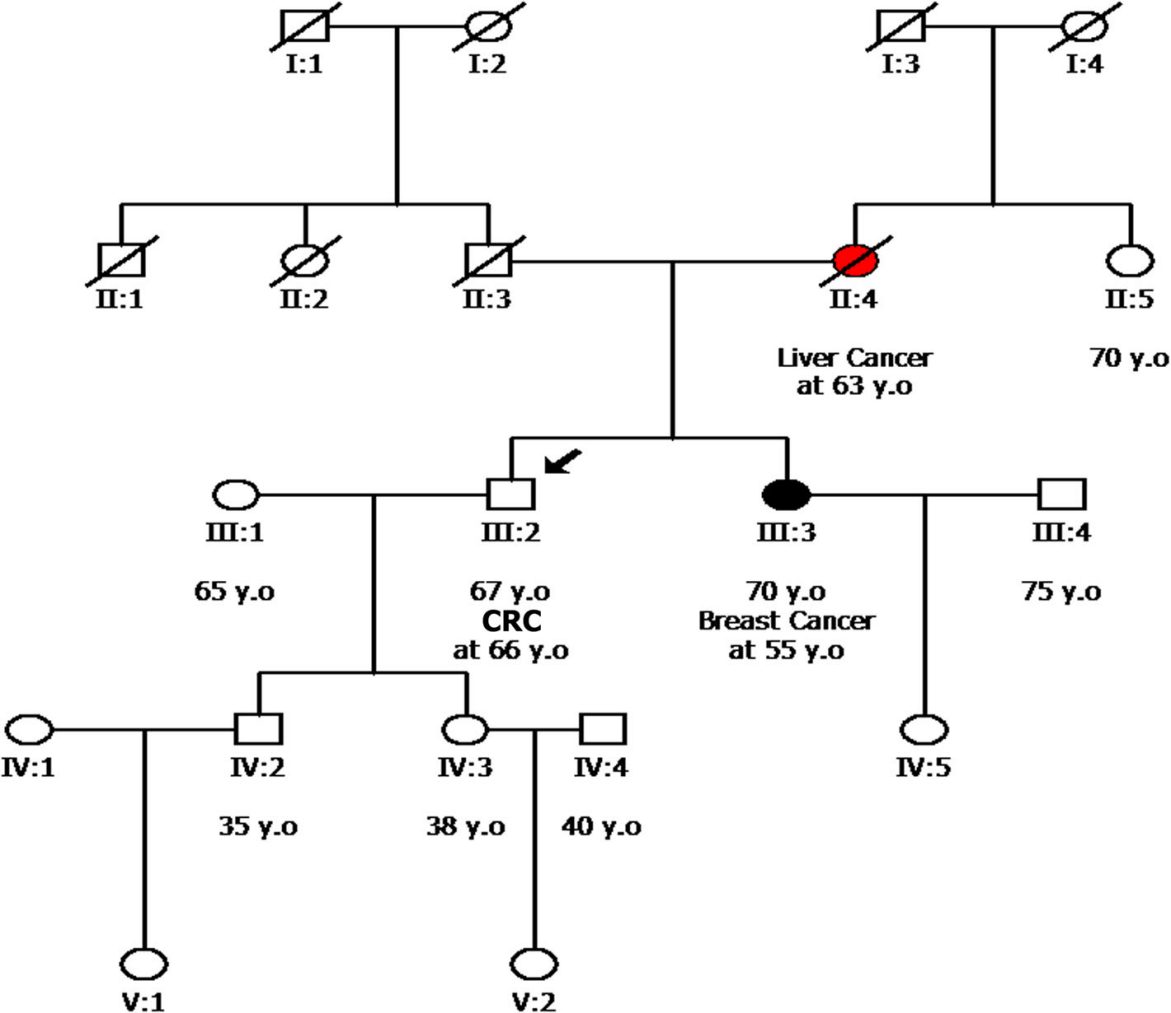
Pedigree of the family of a 67-year old CRC patient

Multi-gene analysis offer the added advantage of identifying pathogenic variants in genes that would normally not be tested based on the proband’s diagnosis. One such case is depicted in the family shown in Fig. 5. The proband (III:2), a 67-year-old male CRC patient, was referred for genetic testing on suspicion of having Lynch Syndrome as the tumor was shown to be microsatellite unstable. However, multi-gene analysis of genomic DNA revealed a pathogenic variant in the *BRCA1* gene.

Considering the patient’s diagnosis and the declared reason for referral, this variant would have been considered an “incidental” finding [53–55] if the proband’s sister (III:3) had not been diagnosed with breast cancer at the age of 55. If testing had been carried out for her the *BRCA1* pathogenic variant would have been considered an “expected” finding rather than an “incidental” one. In reality, what is incidental in this family is the proband’s diagnosis of colorectal cancer, which may have been sporadic. The benefit of the analysis for this family is evident for the proband’s children who have subsequently undergone targeted analysis for the identified *BRCA1* variant. The daughter of the proband (IV:3) who is unaffected at the age of 38 was shown to carry the variant and can now be subjected to the increased surveillance and preventive management recommended for *BRCA1* pathogenic variant unaffected carriers (NCCN guidelines). Targeted analysis of only the Lynch Syndrome genes would have been detrimental in her case.

**Fig. 6:**
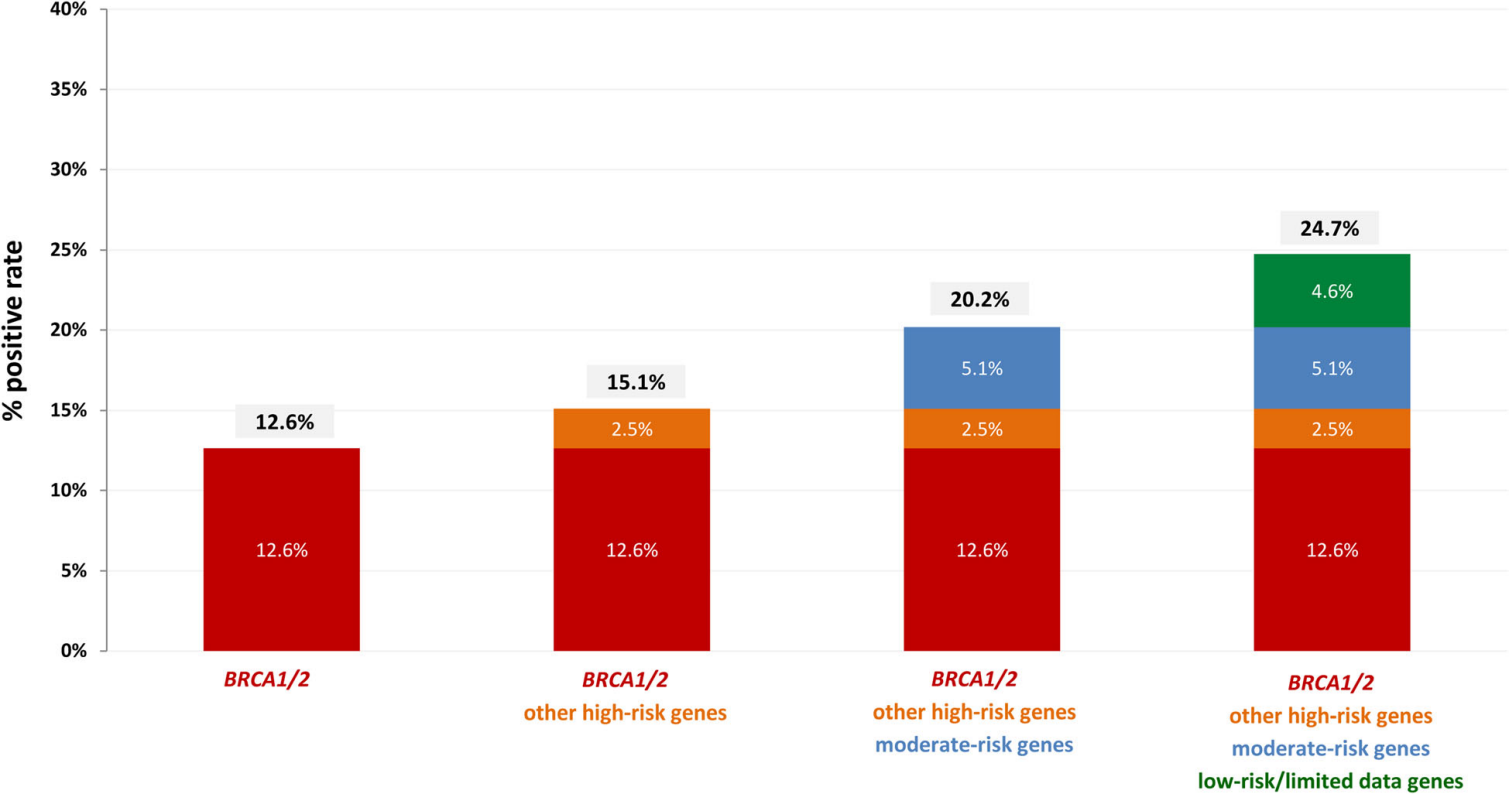
Apportionment of positive results of genetic testing for the 768 individuals with personal history of Breast cancer using 4 different testing scenarios; that of testing the *BRCA1* and *BRCA2* genes only and the three scenarios of using gene panels that include other high-risk, moderate-risk and low-risk genes for breast cancer (Table 1). The percentage in each case corresponds to the number of individuals identified with positive findings

Despite the improved clinical utility of an expanded hereditary cancer gene panel, a higher VUS rate is expected when the number of genes included in a panel increases. In our study, the overall VUS rate for the 1197 individuals was 34.8% (417/1197). The VUS rate of the *BRCA1/2* genes in our cohort was 3.0% (36/1197) (Table 4) with the majority of VUS detected in other high risk genes. (See Additional file 1: Figure S4C). A retrospective analysis of the testing results of breast cancer patients showed that 2.6% of individuals in this group would have received a VUS result if only the *BRCA1/2* genes had been tested and this percentage is increasing when other high, moderate and low risk genes are added to the testing scenario. The highest percentage of VUS in breast cancer patients is detected in low risk genes, or genes with limited/no information for their association with breast cancer (See Additional file 1: Figure S3). However, the scenario where *BRCA1/2* and other high/moderate risk genes for breast cancer are tested shows a positive rate of 20.2% (Fig. 6) with a relative low VUS rate (16.2%).

Nevertheless, present evidence does not suggest that non-clinically significant variant findings be used to modify patient medical management beyond what is indicated by the personal and family history and any other clinically significant findings. However, as described by the term, the clinical significance of these variants is in most cases “uncertain” and not “unknown”, meaning that there is information related to their clinical significance but this information is not yet enough to help us conclude to a definite classification. To address this matter we divided the VUS identified into sub-categories to investigate if there is missing information that could assist towards their classification as likely pathogenic (Class 4) or likely benign (Class 2) variants. We observed that for 76.2% (323/424) of the reported VUS, data is missing for a likely benign classification compared to only 14.2% (60/424) for which data is missing for a likely pathogenic classification. Notably, for only 5.5% (23/424) of VUS the information available is limited and 4.2% (18/424) of VUS are associated with conflicting data preventing an estimation of their clinical significance (See Additional file 1: Figure S5). These findings are further supported by our reclassification results, as all VUS reclassified belonged to the sub-category of VUS missing data for a likely benign classification. Moreover, similar studies have showed that approximately 90–95% of VUS are reclassified because they are being downgraded to likely benign variants [4] showing that the majority of variant reclassification does not impact medical management [56].

Until recently germline variant analysis had a mainly prognostic value in cancer risk assessment. However, such an analysis could also have an impact on treatment selection and clinical management of pathogenic variant carriers. *BRCA1* and *BRCA2* are key proteins of the Homologous Recombination (HR) pathway which is involved in the repair of DNA double-strand breaks. Several studies have shown higher response rates to platinum-based therapy in the presence of *BRCA1*/*2* pathogenic variants [57–59]. Pathogenic variants in these genes are also predictive of response to targeted therapy with PARP inhibitors (PARPi) [60]. PARPi are currently FDA approved for metastatic breast, ovarian and related cancers, mainly in patients with pathogenic variants in the *BRCA1* & *BRCA2* genes [61]. In addition to *BRCA1/ 2*, many other genes encoding HR enzymes are involved in both inherited and acquired cancers and have been associated with PARP inhibitor sensitivity when deficient in vitro or in vivo [62]. In this respect, variants in *ATM, CHEK2, PALB2, RAD51C, RAD51D* and *NBN* have shown the most consistent evidence [60, 63–68]. Thus several ongoing clinical trials are investigating the efficiency of PARPi in cancer patients with both hereditary and somatic variants in genes of the HR pathway. Tumors with common defects in these genes, such as metastatic prostate cancer and pancreatic cancer are the most promising for PARPi upcoming approval.

The present and future clinical implication of the HR gene analysis for treatment selection was seriously considered in our final panel design. Thus, we have included the analysis of 15 HR genes, which are frequently mutated in hereditary cancer (*ATM, BARD1, BRCA1, BRCA2, BRIP1, CHEK1, CHEK2, MRE11A, NBN, PALB2, PTEN, RAD50, RAD51B, RAD51C, RAD51D*). In our cohort, a pathogenic variant in one of the HR genes, was observed in 21.6% (166/768) of the breast cancer patients, while 87.4% (166/190) of the positive findings in breast cancer patients were in one of the HR genes. Overall 18.0% (215/1197) of individuals had a positive finding in a HR gene, or 81.4% (131/161) of pathogenic/likely pathogenic findings were located in an HR gene.

This study was limited by the mode of data collection as the personal and family histories of cancer were ascertained from the self-reported information provided on requisition forms at test uptake. Therefore, some of the information provided may not have been accurate as far as personal and family histories are concerned. Furthermore, approximately 10% of individuals tested provided no information about their personal or family history.

## Conclusions

The advent of NGS technology has enabled the wide use of multi-gene testing in clinical practice. The genetic cause of cancer diagnosis was determined in 22.1% (264/1197) of individuals tested with the vast majority of them (approximately 90% (237/264)) receiving results in genes for which clinical management guidelines are available [46]. These data enable physicians to manage patients based on their genetic background and not only based on personal and family history alone. In addition, identification of pathogenic variants in more than one gene can in some cases explain the diverse tumor types diagnosed in some families.

We anticipate that the accumulation of analyses from mutli-gene testing will increase the available data, shedding further light into the involvement of more genes to cancer predisposition. The contribution of publically available variant databases, where different laboratories can register and interpret, their findings, is of great value [69]. Hence it is recommended for laboratories undertaking multi-gene testing to share their data, and thus contribute to the increase of beneficial information provided by these analyses.

## Additional files

Additional file 1: **Figure S1.** Mutation types for pathogenic/likely pathogenic variants and VUS among the 1197 individuals tested with a hereditary cancer panel A. Mutation types for the 161 unique pathogenic/likely pathogenic variants identified in 264 individuals. B. Mutation types for the 424 unique VUS identified in the 417 individuals. **Figure S2.** Panel testing outcomes and positive results for 768 individuals with personal history of Breast cancer, grouped by gene and gene category based on breast cancer risk (See Additional file 5: Table S4). A. Outcomes of panel testing for the 768 individuals with personal history of Breast cancer. B. Percentages of genes in individuals with positive findings. C. Percentages of gene categories in individuals with positive findings. **Figure S3.** Retrospective analysis of the genetic testing results of the 768 individuals with personal history of Breast cancer with 4 different testing scenarios; that of analyzing the *BRCA1* and *BRCA2* genes only and three additional gene panels that include other high-risk, moderate-risk and low-risk genes for breast cancer (See Additional file 6: Table S5). The percentage in each case corresponds to the number of individuals identified with VUS. **Figure S4.** Statistical analysis of Variants of Uncertain Significance (VUS). A. Number of VUS identified per individual B. Percentage of VUS identified in each gene C. VUS stratified by gene risk category. **Figure S5.** Details about VUS. A. Testing outcomes for individuals tested with a hereditary cancer panel. B. Classification of VUS to sub-categories. (PDF 1131 kb)

Additional file 2: **Table S1.** Frequency of Pathogenic and Likely Pathogenic variants among genes. (PDF 539 kb)

Additional file 3: **Table S2.** List of Pathogenic/ Likely Pathogenic variants. (XLSX 51 kb)

Additional file 4: **Table S3.** Large Genomic Rearrangements (LGRs). (PDF 402 kb)

Additional file 5: **Table S4.** Individuals with 2 Pathogenic/ Likely Pathogenic variants. (PDF 557 kb)

Additional file 6: **Table S5.** List of Variants of Uncertain Significance (VUS). (XLSX 87 kb)

## Data Availability

All data generated or analyzed during this study are included in this published article [and its supplementary information files]. The genomic variants with clinical assertions identified in the current study are available in the ClinVar repository (https://www.ncbi.nlm.nih.gov/clinvar/) and can be searched using the HGVS notation or the accession number for each submitted variant.

## Abbreviations

ACMG: American College of Medical Genetics and Genomics
AMP: Association for Molecular Pathology
CNV: copy number variation
CRC: Colorectal Cancer
ExAC: the Exome Aggregation Consortium database
FAP: Familiar Adenomatous Polyposis
FH: Family History
GME: Great Middle East
gnomAD: the Genome Aggregation Database
HR: Homologous Recombination
LGR: Large Genomic Rearrangement
LM-PCR: Ligation-Mediated Polymerase Chain Reaction
LOVD: Leiden Open-source Variation Database
MAP: *MUTYH* associated polyposis
MID: Multiplex Identifier
MLPA: Multiplex Ligation-dependent Probe Amplification
MMR: DNA Mismatch Repair
MSI: Microsatellite Instability
NCCN: National Comprehensive Cancer Network
NGS: Next Generation Sequencing
PARP: Poly (ADP-ribose) polymerase
PARPi: Poly (ADP-ribose) polymerase inhibitor
PCR: Polymerase Chain Reaction
VUS: Variant of Uncertain Significance
