# Supplementary material for: Analysis of hereditary cancer syndromes by using a panel of genes: novel and multiple pathogenic mutations
Source: BMC Cancer. 2019 Jun 3;19:535. doi: 10.1186/s12885-019-5756-4 (PMC6547505; doi:10.1186/s12885-019-5756-4)
Supplement: Supplementary file 1 — Figure S1. Mutation types for pathogenic/likely pathogenic variants and VUS among the 1197 individuals tested with a hereditary cancer panel A. Mutation types for the 161 unique pathogenic/likely pathogenic variants identified in 264 individuals. B. Mutation types for the 424 unique VUS identified in the 417 individuals. Figure S2. Panel testing outcomes and positive results for 768 individuals with personal history of Breast cancer, grouped by gene and gene category based on breast cancer risk (See Additional file 5: Table S4). A. Outcomes of panel testing for the 768 individuals with personal history of Breast cancer. B. Percentages of genes in individuals with positive findings. C. Percentages of gene categories in individuals with positive findings. Figure S3. Retrospective analysis of the genetic testing results of the 768 individuals with personal history of Breast cancer with 4 different testing scenarios; that of analyzing the BRCA1 and BRCA2 genes only and three additional gene panels that include other high-risk, moderate-risk and low-risk genes for breast cancer (See Additional file 6: Table S5). The percentage in each case corresponds to the number of individuals identified with VUS. Figure S4. Statistical analysis of Variants of Uncertain Significance (VUS). A. Number of VUS identified per individual B. Percentage of VUS identified in each gene C. VUS stratified by gene risk category. Figure S5. Details about VUS. A. Testing outcomes for individuals tested with a hereditary cancer panel. B. Classification of VUS to sub-categories. (PDF 1131 kb) [file 12885_2019_5756_MOESM1_ESM.pdf]

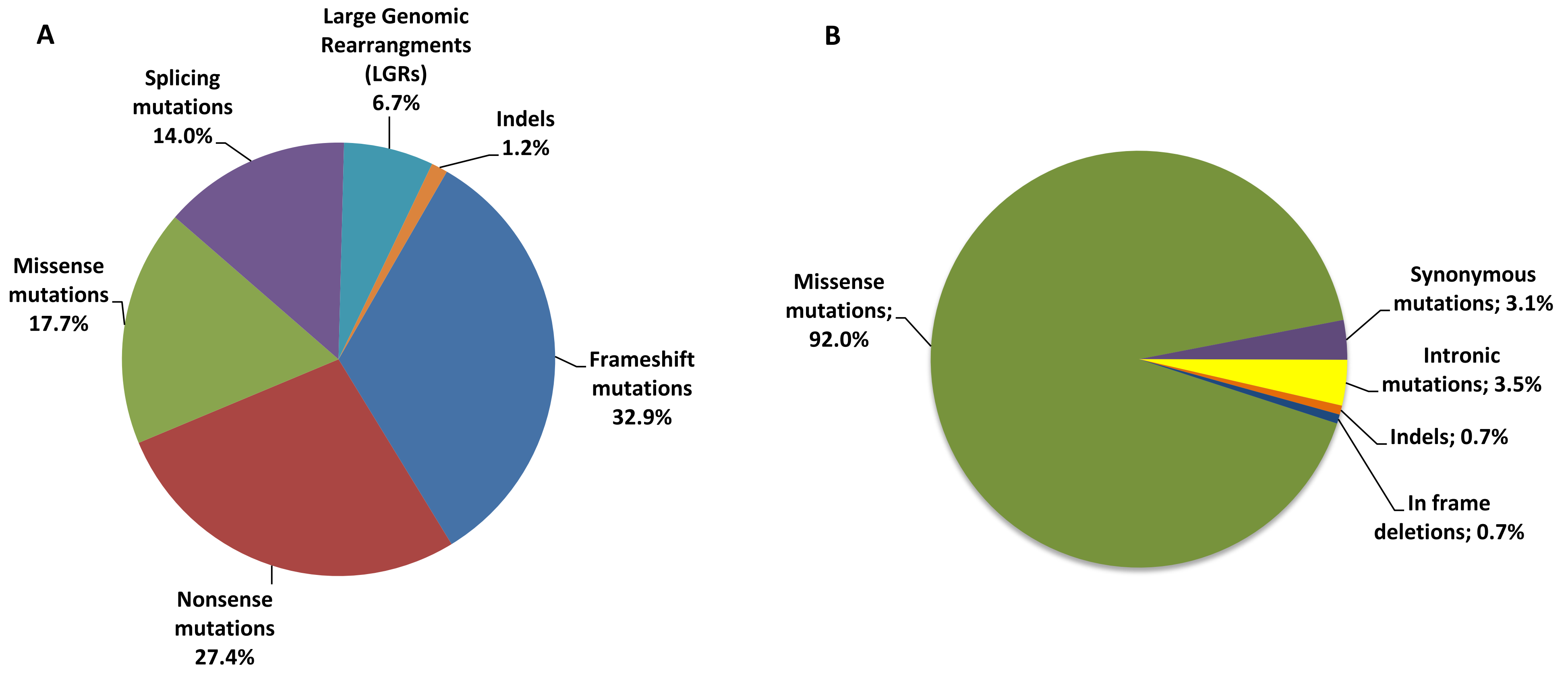

Figure S1 Distribution of mutation types for pathogenic/likely pathogenic variants and VUS among the 1197 individuals tested with a hereditary cancer panel **A.** Mutation types for the 161 unique pathogenic/likely pathogenic variants identified in 264 individuals with positive findings. **B.** Mutation types for the 424 unique VUS identified in the 417 individuals that receive a VUS result.

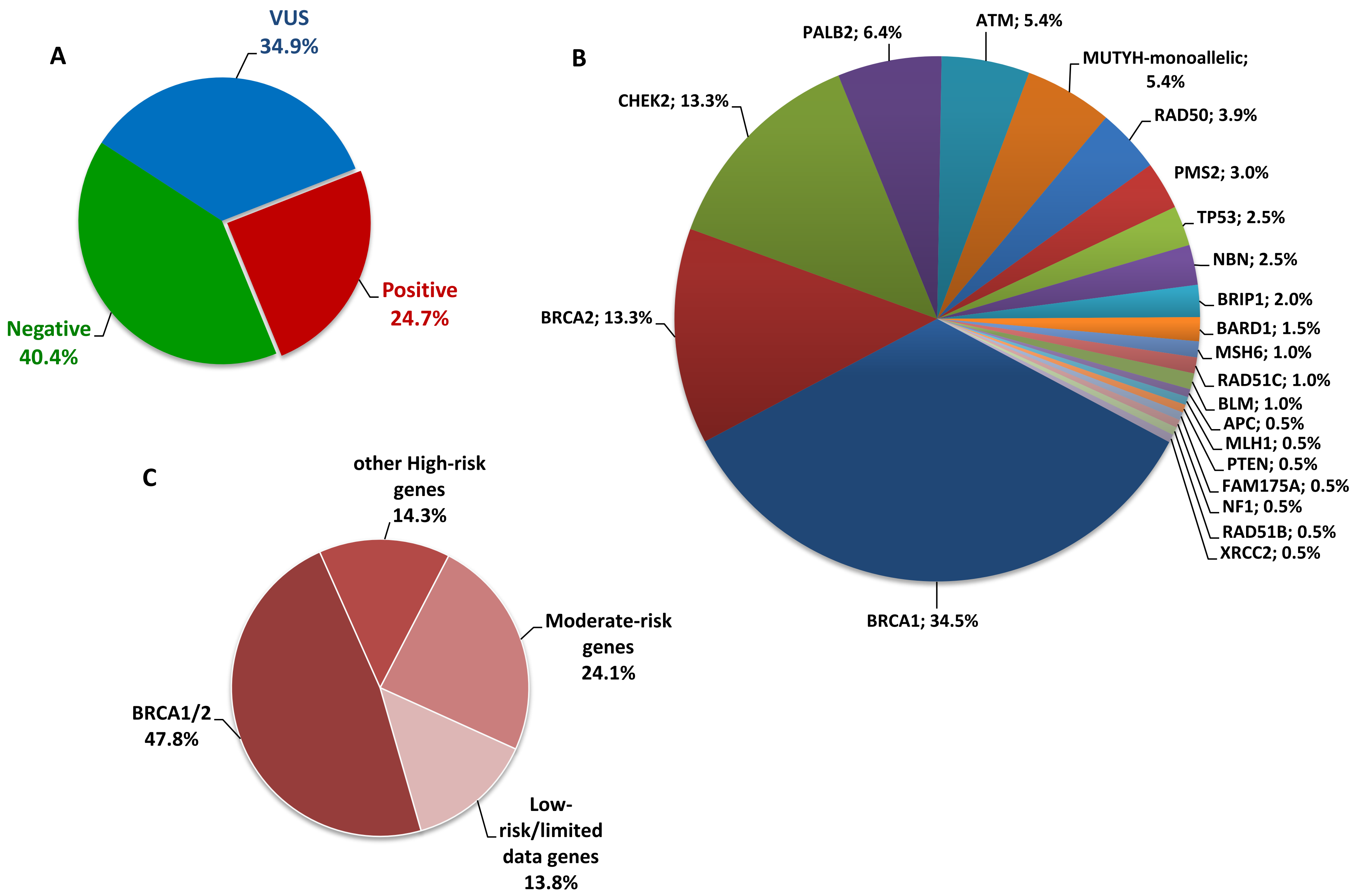

Figure S2 Panel testing outcomes and positive results for the 768 individuals with personal history of Breast cancer, grouped by gene and gene category based on breast cancer risk (Table 1). **A.** Outcomes of panel testing for the 768 individuals with personal history of Breast cancer. **B.** Percentages of genes in individuals with positive findings. **C.** Percentages of gene categories in individuals with positive findings.

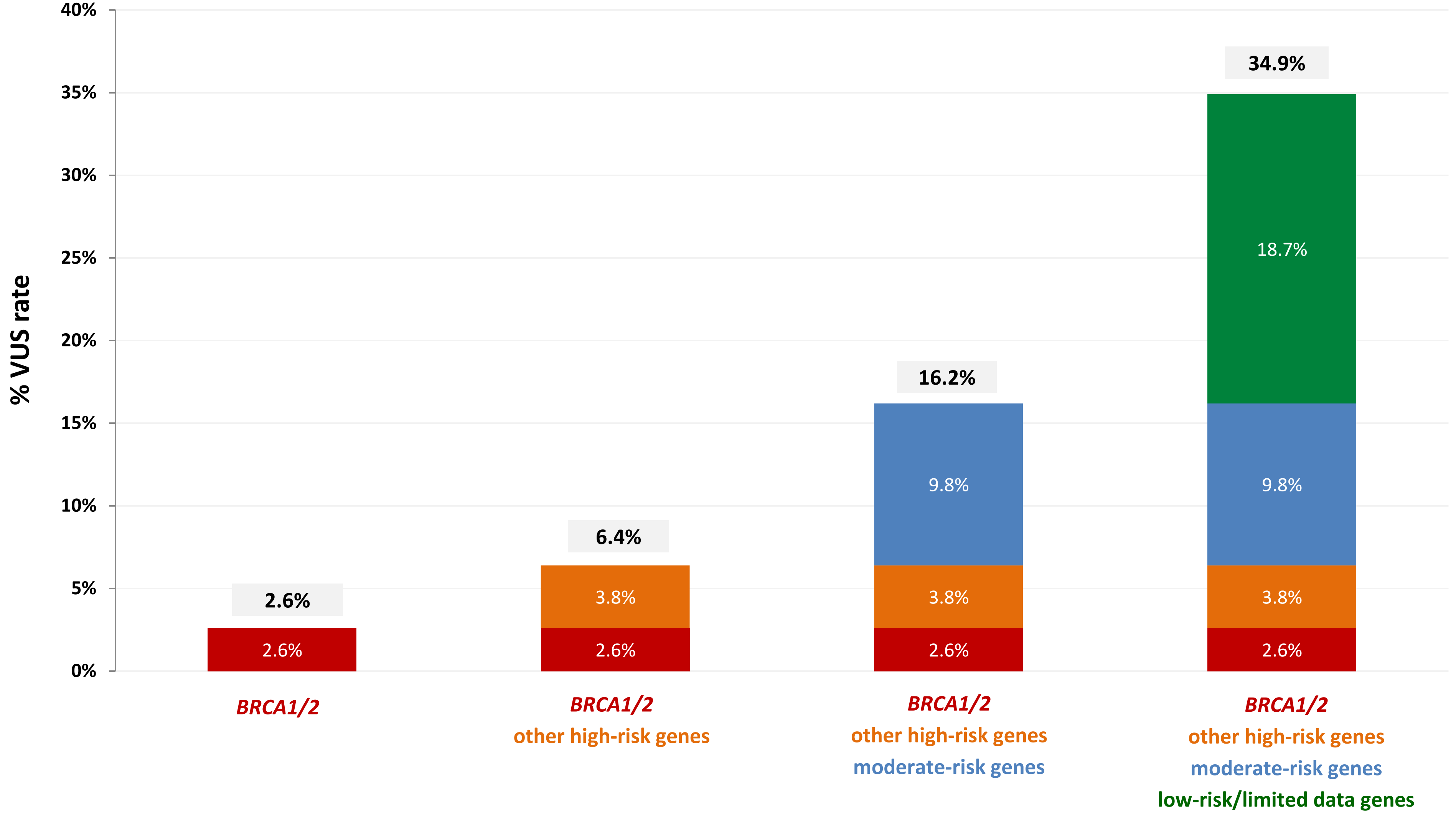

Figure S3 Apportionment of VUS results for the 768 individuals with personal history of Breast cancer in 4 different testing scenarios; that of analyzing the *BRCA1* and *BRCA2* genes only and the three scenarios of using gene panels that include other high-risk, moderate-risk and low-risk genes for breast cancer (Table 1). The percentage in each case corresponds to the number of individuals identified with VUS.

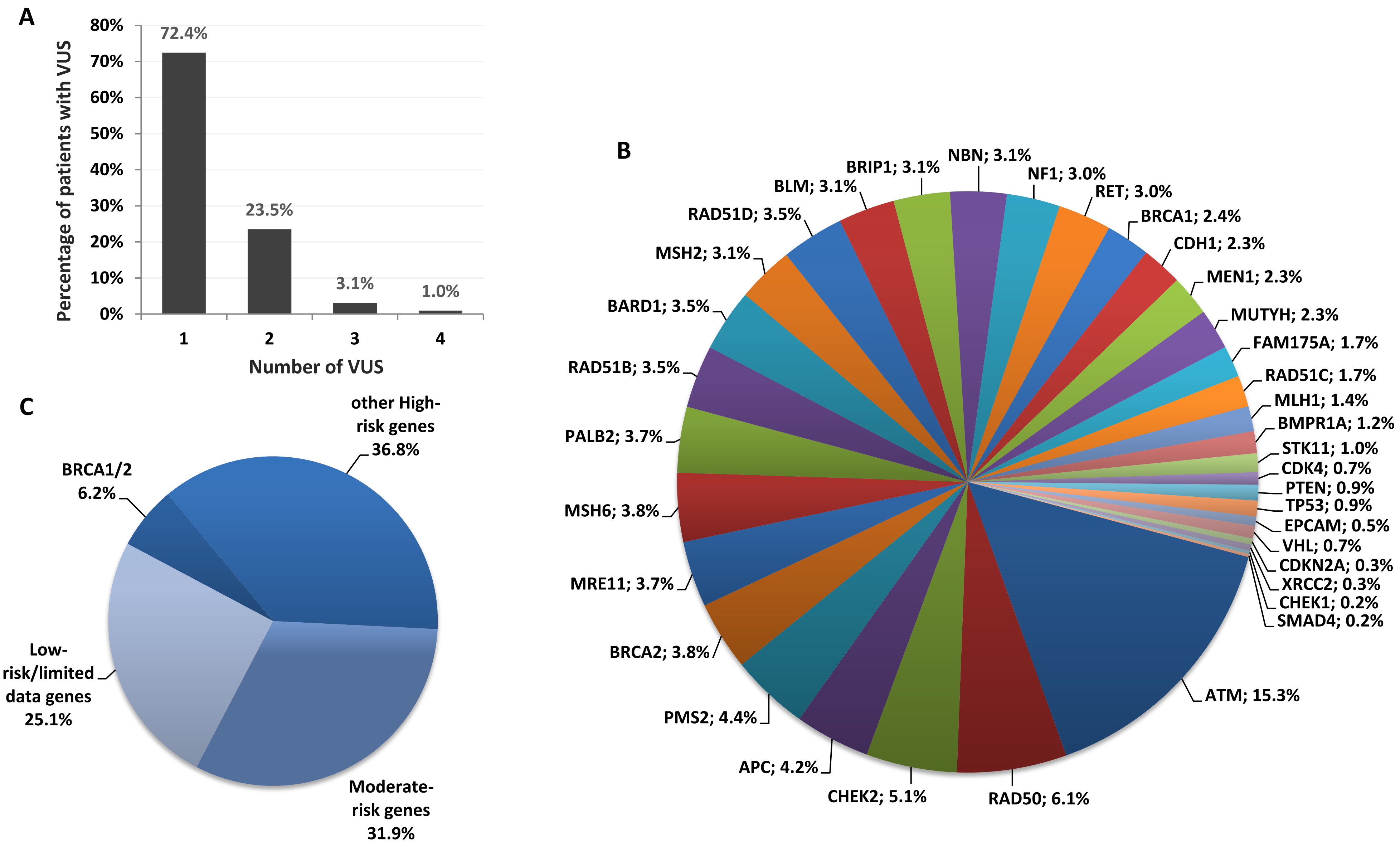

Figure S4 Statistical analysis of Variants of Uncertain Significance (VUS). **A.** Number of VUS identified per individual **B.** Percentage of VUS identified in each gene **C.** VUS stratified by gene risk category.

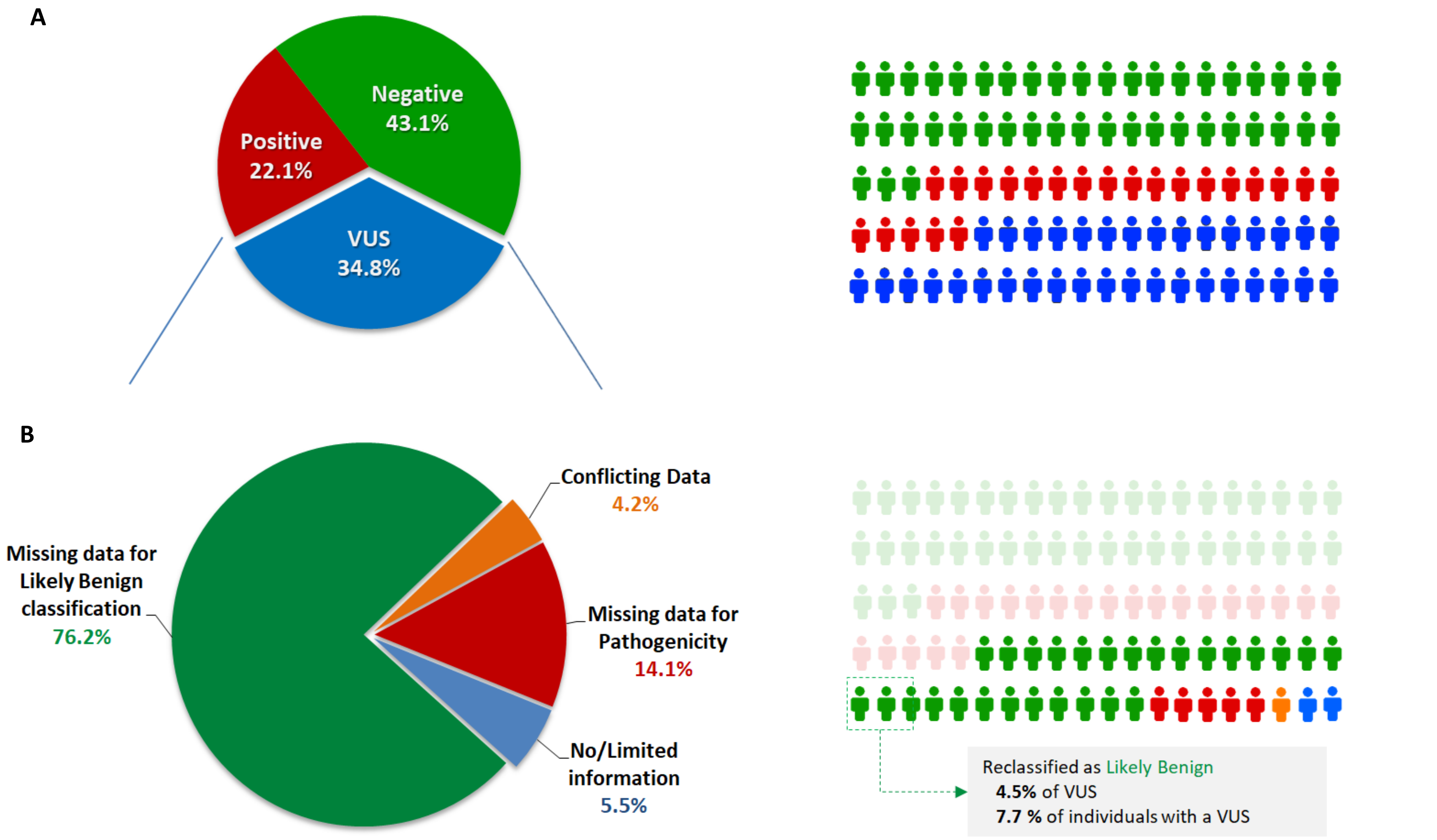

Figure S5 Breakdown of VUS results according to their potential pathogenicity. **A.** Testing outcomes for the 1197 individuals tested with a hereditary cancer panel (left) and illustration on 100 individuals (right). **B.** Classification of VUS to sub-categories taking into account pathogenicity (left) and illustration on 100 individuals (right) including information on the outcomes of reclassifications.
