# Supplementary material for: Analysis of hereditary cancer syndromes by using a panel of genes: novel and multiple pathogenic mutations
Source: BMC Cancer. 2019 Jun 3;19:535. doi: 10.1186/s12885-019-5756-4 (PMC6547505; doi:10.1186/s12885-019-5756-4)
Supplement: Supplementary file 2 — Table S1. Frequency of Pathogenic and Likely Pathogenic variants among genes. (PDF 539 kb) [file 12885_2019_5756_MOESM2_ESM.pdf]

Table S1 Frequency of Pathogenic and Likely Pathogenic variants among genes.

Gene	Total Individuals No. (%)	Affected Individuals No. (%)			Unaffected Individuals No. (%)		
		Breast Cancer	Colorectal Cancer	Ovarian Cancer	FH of Breast Cancer	FH of Colorectal Cancer	FH of Ovarian Cancer
APC	5/771 (0.6)	1/502 (0.2)	2/51 (3.9)	0/23(0.0)	0/72 (0.0)	2/22 (9.1)	0/19 (0.0)
ATM	14/1197 (1.2)	11/768 (1.4)	0/68 (0.0)	0/42 (0.0)	0/103 (0.0)	0/32 (0.0)	1/30 (3.3)
BARD1	5/1197 (0.4)	3/768 (0.4)	0/68 (0.0)	0/42 (0.0)	2/103 (1.9)	0/32 (0.0)	1/30 (3.3)
BLM	2/465 (0.4)	2/297 (0.7)	0/25 (0.0)	0/18 (0.0)	0/36 (0.0)	0/12 (0.0)	0/12 (0.0)
BRCA1	90/1197 (7.5)	70/768 (9.1)	2/68 (2.9)	6/42 (14.3)	3/103 (2.9)	2/32 (6.3)	1/30 (3.3)
BRCA2	36/1197 (3.0)	27/768 (3.5)	0/68 (0.0)	0/42 (0.0)	4/103 (3.9)	0/32 (0.0)	4/30 (13.3)
BRIP1	6/1197 (0.5)	4/768 (0.5)	1/68 (1.5)	0/42 (0.0)	1/103 (1.0)	1/32 (3.1)	1/30 (3.3)
CHEK2	30/1197 (2.5)	27/768 (3.5)	1/68 (1.5)	0/42 (0.0)	1/103 (1.0)	1/32 (3.1)	1/30 (3.3)
EPCAM	1/1197 (0.1)	0/768 (0.0)	1/68 (1.5)	0/42 (0.0)	0/103 (0.0)	0/32 (0.0)	0/30 (0.0)
ABRAXAS1	1/465 (0.2)	1/297 (0.3)	0/25 (0.0)	0/18 (0.0)	0/36 (0.0)	0/12 (0.0)	0/12 (0.0)
MLH1	5/1197 (0.4)	1/768 (0.1)	3/68 (4.4)	0/42 (0.0)	0/103 (0.0)	1/32 (3.1)	0/30 (0.0)
MSH2	5/1197 (0.4)	0/768 (0.0)	3/68 (4.4)	0/42 (0.0)	0/103 (0.0)	0/32 (0.0)	0/30 (0.0)
MSH6	5/1197 (0.4)	2/768 (0.3)	3/68 (4.4)	0/42 (0.0)	0/103 (0.0)	0/32 (0.0)	0/30 (0.0)
MUTYH-monoallelic	21/1197 (1.8)	11/768 (1.4)	5/68 (7.4)	0/42 (0.0)	1/103 (1.0)	0/32 (0.0)	0/30 (0.0)
MUTYH-biallelic	3/1197 (0.3)	0/768 (0.0)	3/68 (4.4)	0/42 (0.0)	0/103 (0.0)	0/32 (0.0)	0/30 (0.0)
NBN	6/1197 (0.5)	5/768 (0.7)	0/68 (0.0)	1/42 (2.4)	0/103 (0.0)	0/32 (0.0)	0/30 (0.0)
NF1	1/527 (0.2)	1/361 (0.3)	0/31 (0.0)	0/19 (0.0)	0/38 (0.0)	0/12 (0.0)	0/12 (0.0)
PALB2	20/1197 (1.7)	13/768 (1.7)	1/68 (1.5)	1/42 (2.4)	1/103 (1.0)	0/32 (0.0)	0/30 (0.0)
PMS2	6/1197 (0.5)	6/768 (0.8)	0/68 (0.0)	0/42 (0.0)	0/103 (0.0)	0/32 (0.0)	0/30 (0.0)
PTEN	1/1197 (0.1)	1/768 (0.1)	0/68 (0.0)	0/42 (0.0)	0/103 (0.0)	0/32 (0.0)	0/30 (0.0)
RAD50	11/1180 (0.9)	8/768 (1.0)	0/68 (0.0)	0/42 (0.0)	1/103 (1.0)	0/32 (0.0)	0/30 (0.0)
RAD51B	1/698 (0.1)	1/471 (0.2)	0/47 (0.0)	0/21 (0.0)	0/68 (0.0)	0/18 (0.0)	0/18 (0.0)
RAD51C	2/1197 (0.2)	2/768 (0.3)	0/68 (0.0)	0/42 (0.0)	0/103 (0.0)	0/32 (0.0)	0/30 (0.0)
RET	2/763 (0.3)	0/502 (0.0)	1/47 (2.1)	0/23 (0.0)	0/72 (0.0)	0/21 (0.0)	0/19 (0.0)
STK11	1/1197 (0.1)	0/768 (0.0)	0/68 (0.0)	0/42 (0.0)	0/103 (0.0)	0/32 (0.0)	0/30 (0.0)
TP53	6/1197 (0.5)	5/768 (0.7)	0/68 (0.0)	0/42 (0.0)	0/103 (0.0)	0/32(0.0)	0/30 (0.0)
VHL	1/763 (0.0)	0/502 (0.0)	0/47 (0.0)	0/23 (0.0)	0/72 (0.0)	0/21 (0.0)	0/19 (0.0)
XRCC2	1/465 (0.2)	1/297 (0.3)	0/25 (0.0)	0/18 (0.0)	0/36 (0.0)	0/12 (0.0)	0/12 (0.0)
