# Supplementary material for: Analysis of hereditary cancer syndromes by using a panel of genes: novel and multiple pathogenic mutations
Source: BMC Cancer. 2019 Jun 3;19:535. doi: 10.1186/s12885-019-5756-4 (PMC6547505; doi:10.1186/s12885-019-5756-4)
Supplement: Supplementary file 4 — Table S3. Large Genomic Rearrangements (LGRs). (PDF 402 kb) [file 12885_2019_5756_MOESM4_ESM.pdf]

Table S3 Large Genomic Rearrangements (LGRs)

GENE	HGVS nomenclature	Other nomenclature	Traditional nomenclature	# detected
BRCA1	NM_007294:c.(?_-1)_(80+1_81-1)del	deletion of exons 1-2		2
BRCA1	NM_007294:c.(441+1_442-1)_(547+1_548-1)del	deletion of exon 7	deletion of exon 8	1
BRCA1	NM_007294:c.(5074+1-5075-1)_(5193+1_5194-1)del	deletion of exons 17-18	deletion of exons 18-19	1
BRCA1	NM_007294:c.(5467+1_5468-1)-(*1_?)del	deletion of exon 23	deletion of exon 24	4
BRCA1	NM_007294:c.(80+1_81-1)_(134+1_135-1)del	deletion of exon 3		1
BRCA2	NM_000059:c.(6841+1_6842-1)_(7007+1_7008-1)del	deletion of exons 12-13		1
CHEK2	NM_007194:c.(908+1_909-1)_(1095+1_1096-1)del	deletion of exons 9-10		2
EPCAM/MSH2	NM_002354:c.(903+1_904-1)_(*1_?)del AND NM_000251:c. (?-1)_(1276+1_1277-1)del	deletion of 3' UTR of EPCAM and deletion of exons 1-7 of MSH2		1
MLH1	NM_000249:c.(453+1_454-1)_(545+1_546-1)del	deletion of exon 6		1
MSH2	NM_000251:c.(1661+1_1662-1)_(*1_?)del	deletion of exons 11-16		1
PMS2	NM_000535:c.(?-1)_(*1_?)del	deletion of exons 1-15 (entire PSM2 gene)		1
