# Supplementary material for: Analysis of hereditary cancer syndromes by using a panel of genes: novel and multiple pathogenic mutations
Source: BMC Cancer. 2019 Jun 3;19:535. doi: 10.1186/s12885-019-5756-4 (PMC6547505; doi:10.1186/s12885-019-5756-4)
Supplement: Supplementary file 5 — Table S4. Individuals with 2 Pathogenic/ Likely Pathogenic variants. (PDF 557 kb) [file 12885_2019_5756_MOESM5_ESM.pdf]

Table S4 Individuals with 2 Pathogenic/ Likely Pathogenic variants.

#	Gene	cDNA	Protein	HGVS nomenclature	Classification	Personal History of cancer
1	*MSH2*	Deletion (exons 11-16)			Pathogenic	Endometrium
	PALB2	c.757_758delCT	p.Leu253Ilefs*3		Pathogenic	
2	*BRCA1*	c.181T>G	p.Cys61Gly		Pathogenic	Breast
	CHEK2	c.470T>C	p.Ile157Thr		Pathogenic (low penetrance)	
3	*MSH6*	c.2136delG	p.Asp713Ilefs*23		Pathogenic	Breast
	MUTYH	c.1187G>A	p.Gly396Asp		Pathogenic	
4	*CHEK2*	c.1232G>A	p.Trp411*		Pathogenic	Breast
	PMS2	c.(?_-87)_(*160_?)del			Pathogenic	
5	*MUTYH*	c.536A>G	p.Tyr179Cys		Pathogenic	CRC
	MUTYH	c.1437_1439delGGA	p.Glu479_Glu480delinsGlu		Pathogenic	
6	*MSH2*	c.2131C>T	p.Arg711*		Pathogenic	Endometrium and CRC
	MUTYH	c.1187G>A	p.Gly396Asp		Pathogenic	
7	*BRCA1*	c.737delT	p.Leu246*		Pathogenic	Breast
	CHEK2	c.470T>C	p.Ile157Thr		Pathogenic (low penetrance)	
8	*MSH2*	c.2131C>T	p.Arg711*		Pathogenic	CRC
	MUTYH	c.1187G>A	p.Gly396Asp		Pathogenic	
9	*MSH2*	c.2131C>T	p.Arg711*		Pathogenic	Polyps in bile, uterus, stomach
	MUTYH	c.1187G>A	p.Gly396Asp		Pathogenic	
10	*BLM*	c.1642C>T	p.Gln548*		Pathogenic	Breast
	CHEK2	c.1283C>T	p.Ser428Phe		Pathogenic	
11	*MLH1*	c.1409+1G>A			Likely Pathogenic	CRC and cholangioma
	MUTYH	c.734G>A	p.Arg245His		Pathogenic	
12	*BLM*	c.1642C>T	p.Gln548*		Pathogenic	Breast
	BRCA1	c.3700_3704delGTAAA	p.Val1234_Asn1235?fs		Pathogenic	

13	*BRCA2*	c.9154C>T	p.Arg3052Trp	Pathogenic	Breast
	MUTYH	c.1187G>A	p.Gly396Asp	Pathogenic	
14	*FAM175A*	c.1106dupG	p.Ser370Ilefs*2	Pathogenic	Triple Negative Breast
	RAD50	c.3779_3791dupGTAACTTCCAGCT	p.Leu1265*	Pathogenic	
15	*BRCA1*	c.5266dupC	p.Gln1756Profs*74	Pathogenic	Not Available (NA)
	RAD50	c.326_329delCAGA	p.Thr109Asnfs*20	Pathogenic	
16	*NBN*	c.657_661delACAAA	p.Lys219Asnfs*16	Pathogenic	Breast
	PMS2	c.1261C>T	p.Arg421*	Pathogenic	
17	*BRCA1*	c.843_846delCTCA	p.Ser282Tyrfs*15	Pathogenic	Breast
	CHEK2	c.470T>C	p.Ile157Thr	Pathogenic (low penetrance)	
18	*CHEK2*	c.470T>C	p.Ile157Thr	Pathogenic (low penetrance)	Breast
	CHEK2	c.499G>A	p.Gly167Arg	Likely Pathogenic	
19	*MUTYH*	c.734G>A	p.Arg245His	Pathogenic	Breast
	PALB2	c.2257C>T	p.Arg753*	Pathogenic	
20	*MUTYH*	c.536A>G	p.Tyr179Cys	Pathogenic	Breast
	RAD51C	c.905-2A>G		Pathogenic	
21	*BRCA1*	c.5212G>A	p.Gly1738Arg	Pathogenic	Breast
	BRIP1	c.2947delA	p.Ile983Leufs*2	Likely Pathogenic	
22	*MUTYH*	c.1437_1439delGGA	p.Glu479_Glu480delinsGlu	Pathogenic	CRC
	RET	c.2410G>A	p.Val804Met	Pathogenic	
23	*BRCA2*	c.9371A>T	p.Asn3124Ile	Likely Pathogenic	Breast
	CHEK2	c.902delT	p.Leu301Trpfs*3	Pathogenic	
24	*APC*	c.4391_4394delAGAG	p.Glu1464Valfs*8	Pathogenic	Polyps
	PALB2	c.3340C>T	p.Gln1114*	Pathogenic	
25	*BRIP1*	c.2575+1G>A		Likely Pathogenic	CRC
	MLH1	c.(453+1_454-1)_(545+1_546-1)del		Pathogenic	
